# VIT-2763, a ferroportin-1 inhibitor, modulates osteoblast and osteoclast functions in hemizygous β^IVSII-654^ knock-in thalassemic mice with osteopenia

**DOI:** 10.1038/s41420-026-03273-x

**Published:** 2026-07-31

**Authors:** Ratchaneevan Aeimlapa, Jarinthorn Teerapornpuntakit, Jirawan Thongbunchoo, Pathnaree Wattano, Jiraporn Panmanee, Saovaros Svasti, Narattaphol Charoenphandhu

**Affiliations:** 1 Center of Calcium and Bone Research (COCAB), Faculty of Science, Mahidol University, Bangkok, Thailand; 2 Department of Physiology, Faculty of Science, Mahidol University, Bangkok, Thailand; 3 Physiology Division, Preclinical Science, Faculty of Medicine, Thammasat University, Pathum Thani, Thailand; 4 Research Center for Neuroscience, Institute of Molecular Biosciences, Mahidol University, Nakhon Pathom, Thailand; 5 Thalassemia Research Center, Institute of Molecular Biosciences, Mahidol University, Nakhon Pathom, Thailand; 6 Institute of Molecular Biosciences, Mahidol University, Nakhon Pathom, Thailand; 7 The Academy of Science, The Royal Society of Thailand, Bangkok, Thailand

**Keywords:** Bone, Cellular imaging

## Abstract

Iron overload is a serious complication of thalassemia that affects various organ functions. Although iron chelators effectively alleviate iron overload, they have some adverse effects on bone structure. VIT-2763, an oral ferroportin-1 inhibitor, has been recently reported to reduce iron overload and improve ineffective erythropoiesis in β-thalassemia. Since iron is required for osteoblast and osteoclast functions, VIT-2763 was hypothesized to modulate bone metabolism. Herein, we used hemizygous β^IVSII-654^ knock-in thalassemic (B654) mice as a model of β-thalassemia with skeletal defects including trabecular bone loss and cortical thinning. At the cellular level, mRNA expression of bone formation-related genes (e.g., Runx2, ALP, osteocalcin) was upregulated in B654-derived primary osteoblasts. The mRNA expression of those bone formation-related genes was negatively correlated with ferroportin-1 expression in primary osteoblasts. In primary osteoclasts, the mRNA expression of mature osteoclast-specific genes was positively correlated with ferroportin-1 expression, suggesting an involvement of ferroportin-1 in regulating bone formation and resorption. We further showed that VIT-2763 effectively suppressed ferroportin-1 protein expression in both primary osteoblasts and osteoclasts. VIT-2763 decreased viability of primary osteoblasts without affecting osteoblast-specific gene expression and bone mineralization. Interestingly, VIT-2763 biphasically regulated osteoclastogenesis, particularly in early osteoclast differentiation, and attenuated osteoclast-mediated bone resorption in vitro. Quantitative analysis of three-dimensional (3D) osteoclastic hologram images found that VIT-2763 significantly altered cellular properties (e.g., mean refractive index) of primary osteoclasts in iron-loaded conditions. In conclusion, modulation of ferroportin-1 expression and cellular functions by VIT-2763 potentially inhibited osteoclast-mediated bone resorption. It may be a novel anti-resorptive agent for treating iron-induced osteopathy in β-thalassemia.

**Figure:**
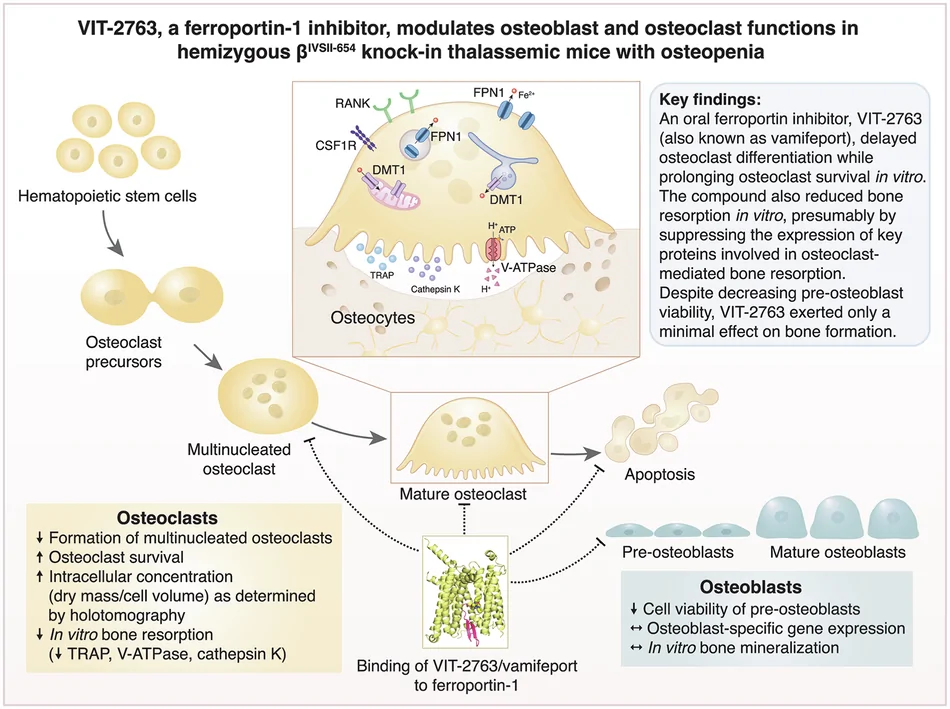

## Introduction

Osteopenia is one of thalassemic complications that impacts quality of life. Thalassemia-associated bone defects could result from chronic inflammation, calcium imbalance, endocrine dysregulation, and iron overload [1]. Iron overload frequently presents in thalassemic patients with regular blood transfusion, non-transfusion-dependent thalassemia with occasional blood transfusion, and hereditary hemochromatosis. Iron overload was reported to have detrimental effects on bone by decreasing osteoblast-mediated bone formation and increasing osteoclast-mediated bone resorption [2]. Thalassemic patients with regular blood transfusion usually exhibited low bone mineral density (BMD) and bone fragility [3]. Although iron chelation therapy effectively alleviates iron overload, the information about iron chelators on bone health has been limited.

Under physiological conditions, plasma iron is primarily regulated by liver-derived hepcidin. Hepcidin downregulated ferroportin-1, an iron exporter, expression in enterocytes, hepatocytes, and macrophages, leading to decreased intestinal iron absorption and iron export from the cells [4, 5]. However, low hepcidin levels appeared in β-thalassemia, that was likely due to increased erythroferrone levels, a negative regulator of hepcidin [6, 7]. Consequently, iron hyperabsorption and elevated serum iron levels partly contributed to iron overload in β-thalassemia [8, 9].

Ferroportin-1 plays a vital role in the regulation of whole-body iron homeostasis. In bone marrow-derived macrophages, ferroportin-1 protein was mostly localized in cytoplasm as intracellular vesicles under basal condition [10, 11]. Ferroportin-1 protein was highly expressed on plasma membrane during iron-loaded cells, while its overall expression was reduced in iron-deficient cells [10]. Compensatory upregulation of ferroportin-1 in response to high iron was suggested to prevent iron-induced oxidative stress in the cells.

Currently, an oral ferroportin-1 inhibitor called VIT-2763—also known as vamifeport—has been discovered. Like hepcidin but with less efficacy, VIT-2763 attenuated ferroportin-1 protein expression by inducing ferroportin-1 internalization and degradation. In addition to alleviating iron overload, VIT-2763 also improved ineffective erythropoiesis and anemia in β-thalassemia and β-thalassemia with blood transfusion [12, 13]. Furthermore, VIT-2763 efficacy still persisted even in combined treatment with iron chelator deferasirox [14]. Clinical study supported that VIT-2763 had no severe adverse effects in healthy individuals [15]. Although ferroportin-1 was widely expressed by bone cells and precursors of bone cells [16–18], there is no report on whether VIT-2763 impacts bone metabolism, particularly in β-thalassemic condition. The present study aimed to investigate the effects of VIT-2763 on the cellular functions of bone and the potential use of VIT-2763, a newly developed oral ferroportin-1 inhibitor, for the treatment of thalassemic osteopathy. Our study revealed that VIT-2763 did not significantly influence osteoblast-mediated bone formation. On the other hand, osteoclastogenesis and osteoclastic bone resorption were markedly abolished by VIT-2763. The novelty of this study lies in demonstrating the promising effects of this drug and highlighting ferroportin-1 as a potential target for the treatment of iron-related osteopathy.

## Results

### B654 mice exhibited trabecular bone loss, cortical thinning, and iron imbalance

**Fig. 1:**
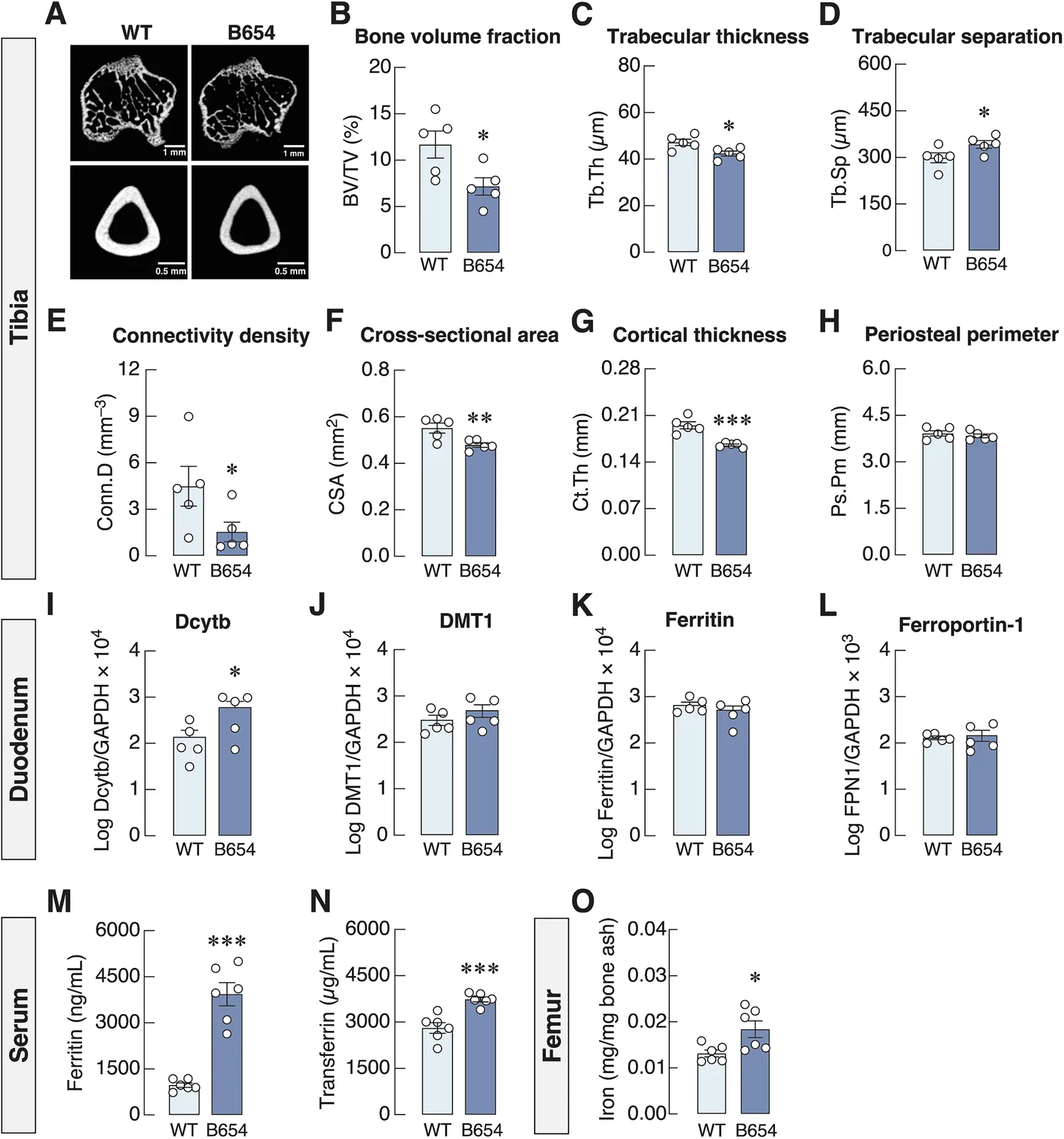
Trabecular bone loss, cortical thinning, and iron imbalance caused by β-thalassemia. **A** representative 3D bone microstructure of tibial metaphysis and diaphysis of wild-type (WT) and hemizygous β^IVSII-654^ knock-in thalassemic (B654) mice. Microstructural analysis of tibia was determined by micro-computed tomography (μCT). **B** Trabecular bone volume fraction (BV/TV), **C** trabecular thickness (Tb.Th), **D** trabecular separation (Tb.Sp), **E** connectivity density (Conn.D), **F** cross-sectional area (CSA), **G** cortical thickness (Ct.Th), **H** periosteal perimeter (Ps.Pm) were assessed by Bone J (ImageJ plugin). mRNA expression of iron transporter genes, i.e., **I** duodenal cytochrome b (Dcytb), **J** divalent metal transporter 1 (DMT1), **K** ferritin, **L** ferroportin-1, and serum levels of **M** ferritin, **N** transferrin in 16-week-old WT and B654 mice. **O** Femoral iron levels in 28-week-old WT and B654 mice. Data expressed as mean ± SE (*n* = 5–6). *P < 0.05, **P < 0.01, ***P < 0.001 vs. age-matched WT by unpaired one-tailed Student’s *t* test.

It has been reported that a C → T point mutation at nucleotide 654 of the intron 2 of the β-globin gene results in thalassemia intermedia [19, 20]. The present study used B654 mice as a model of β-thalassemia with mild iron overload, supporting by complete blood count (CBC) findings (Supplementary Table S2). μCT analysis revealed that B654 mice had less trabecular bone as indicated by decreases in bone volume fraction (BV/TV), trabecular thickness (Tb.Th), and connectivity density (Conn.D), while increasing trabecular separation (Tb.Sp) (Fig. 1). Moreover, the decreased cross-sectional area and cortical thickness, but not periosteal perimeter (Fig. 1), supported the presence of cortical thinning in B654 mice. To investigate the possible mechanism of iron overload in these mice, iron hyperabsorption was hypothesized as a cause of iron overload. Generally, the duodenum is a major site of intestinal iron absorption. Ferric iron is reduced to ferrous iron by Dcytb prior to iron entry via DMT1. Intracellular iron binds to ferritin as an intracellular iron storage or is exported via ferroportin-1 [21]. The mRNA expression level of Dcytb, but not DMT1, ferritin, or ferroportin-1, was significantly increased in the B654 duodenum (Fig. 1I–L). Thus, upregulation of the duodenal iron transporter gene may partly contribute to iron overload in the B654 mouse model. Elevation of serum ferritin and transferrin levels (Fig. 1M, N) and femoral iron (Fig. 1O) indicated an increase in systemic iron levels and bone iron deposition in B654 mice.

### Compensatory upregulation of bone formation-related genes was associated with decreased ferroportin-1 expression

**Fig. 2:**
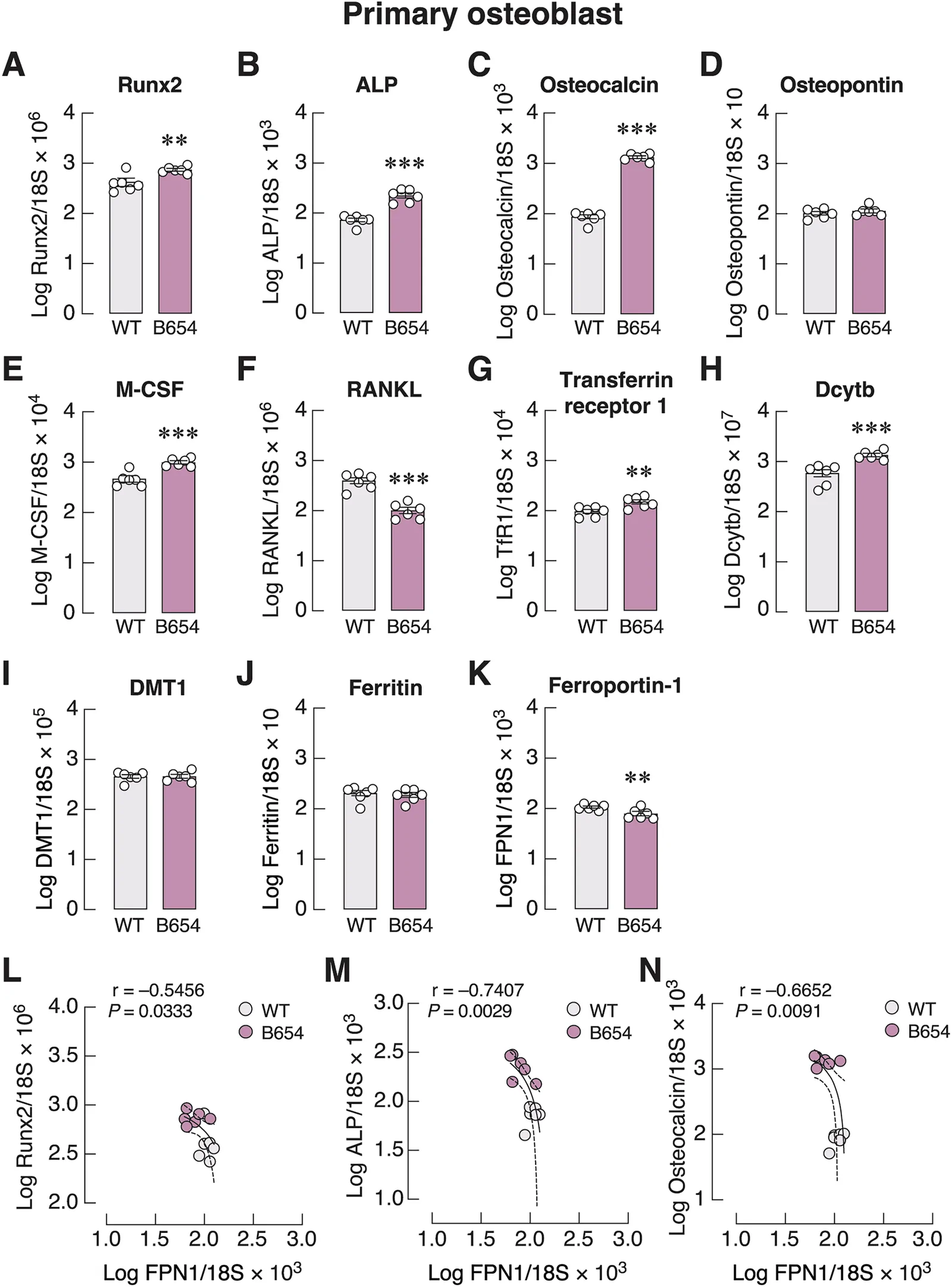
Association of upregulated bone formation-related genes in β-thalassemia-derived osteoblasts with decreased ferroportin-1 expression. The mRNA expression of osteoblast-specific genes, i.e., **A** Runx2, **B** alkaline phosphatase (ALP), **C** osteocalcin, **D** osteopontin, osteoclastogenic factors, i.e., **E** macrophage-colony stimulating factor (M-CSF), **F** receptor activator of nuclear factor-κB ligand (RANKL), and iron transporter genes, i.e., **G** transferrin receptor 1 (TfR1), **H** duodenal cytochrome b (Dcytb), **I** divalent metal transporter 1 (DMT1), **J** ferritin, **K** ferroportin-1 in primary osteoblasts derived from wild-type (WT) and hemizygous β^IVSII-654^ knock-in thalassemic (B654) mice. Data expressed as mean ± SE (*n* = 6). Correlation plot between mRNA expression of ferroportin-1 and **L** Runx2, **M** ALP, **N** osteocalcin in primary osteoblasts. Dashed line represents the 95% confident bands of the best-fit line. r Pearson correlation coefficient. *P* *p*-value of the correlation. **P < 0.01, ***P < 0.001 vs. age-matched WT by unpaired one-tailed Student’s *t* test.

Previous study revealed that trabecular bone loss in B654 mice was partly due to decreased bone formation and increased bone resorption [22]. However, the cellular mechanism of bone changes has not been investigated. We herein demonstrated that mRNA expression levels of most bone formation-related genes (i.e., Runx2, ALP, osteocalcin), osteoclastogenic factor (i.e., M-CSF), and some iron transport system (i.e., transferrin receptor 1, and Dcytb) were upregulated, while ferroportin-1 mRNA expression was significantly downregulated in B654 osteoblasts (Fig. 2). Correlation analyses showed the negative correlation between ferroportin-1 and bone formation-related genes (Fig. 2L–N), suggesting an involvement of ferroportin-1 in the regulation of bone formation.

### Decrease in differentiation potential of B654-derived osteoclast precursors

**Fig. 3:**
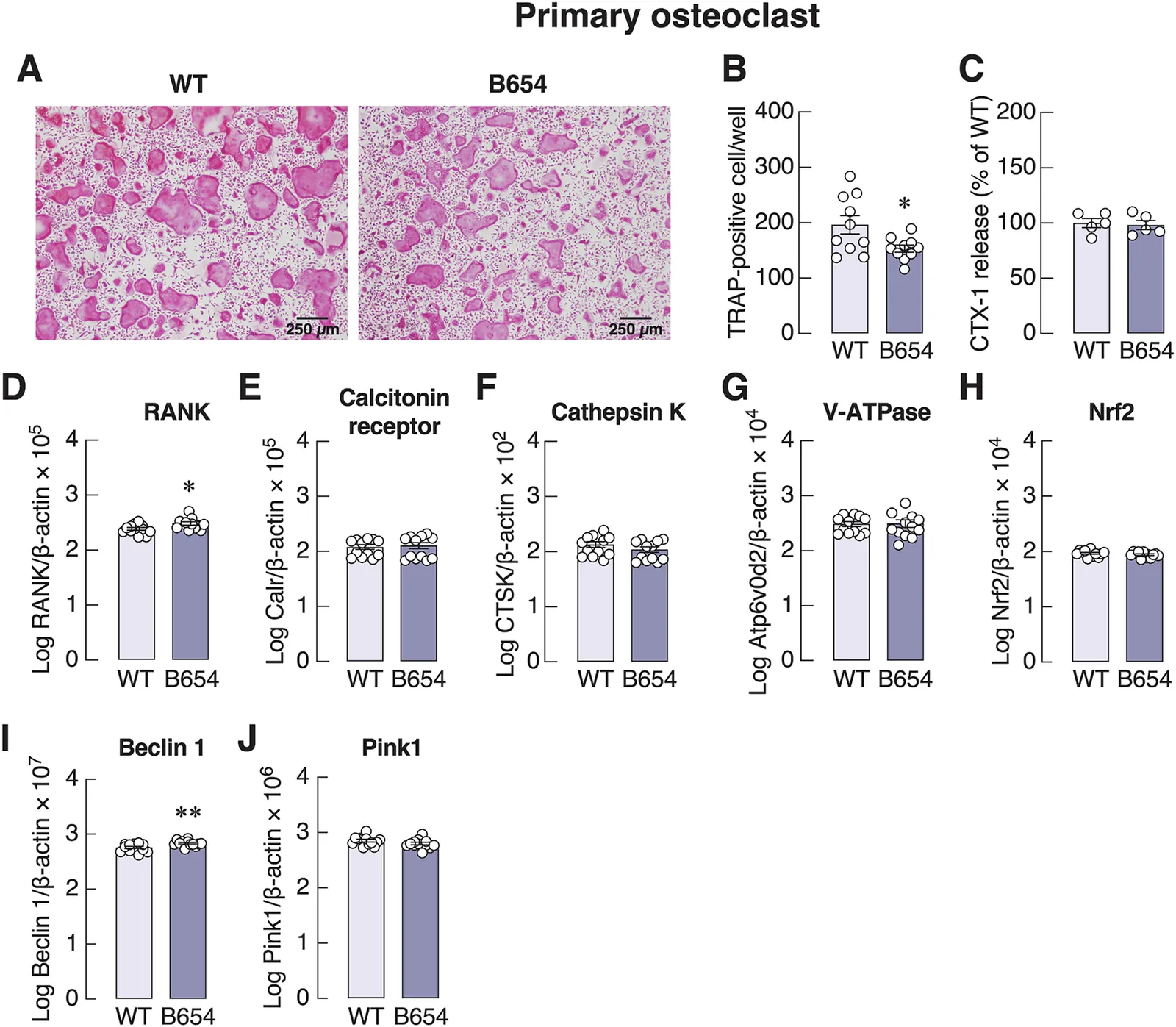
Lower differentiation potential but preserved resorptive activity in β-thalassemia-derived osteoclast precursors. Representative images of TRAP-positive multinucleated osteoclasts after culture for 8 days. Scale bars, 250 μm (×4 magnification). **B** Number of TRAP-positive cells were manually counted. **C** The levels of released C-terminal telopeptides of type I collagen (CTX-1) in culture media after culture bone marrow-derived osteoclasts on bone slices at day 13. mRNA expression of osteoclast-specific genes, i.e., **D** receptor for receptor activator of nuclear factor-κB ligand (RANK), **E** calcitonin receptor (Calr), **F** cathepsin K (CTSK), **G** vacuolar ATPase H^+^ transporting V0 subunit D2 (ATP6v0d2 or V-ATPase), transcription factor, i.e., **H** nuclear factor erythroid 2-related factor 2 (Nrf2), and mitophagy-related genes, i.e., **I** Beclin 1, **J** PTEN-induced kinase 1 (Pink1) in primary osteoclasts derived from bone marrow cells of wild-type (WT) and hemizygous β^IVSII-654^ knock-in thalassemic (B654) mice. Data expressed as mean ± SE (*n* = 5–12). *P < 0.05, **P < 0.01 vs. age-matched WT by unpaired one-tailed Student’s *t* test.

**Fig. 4:**
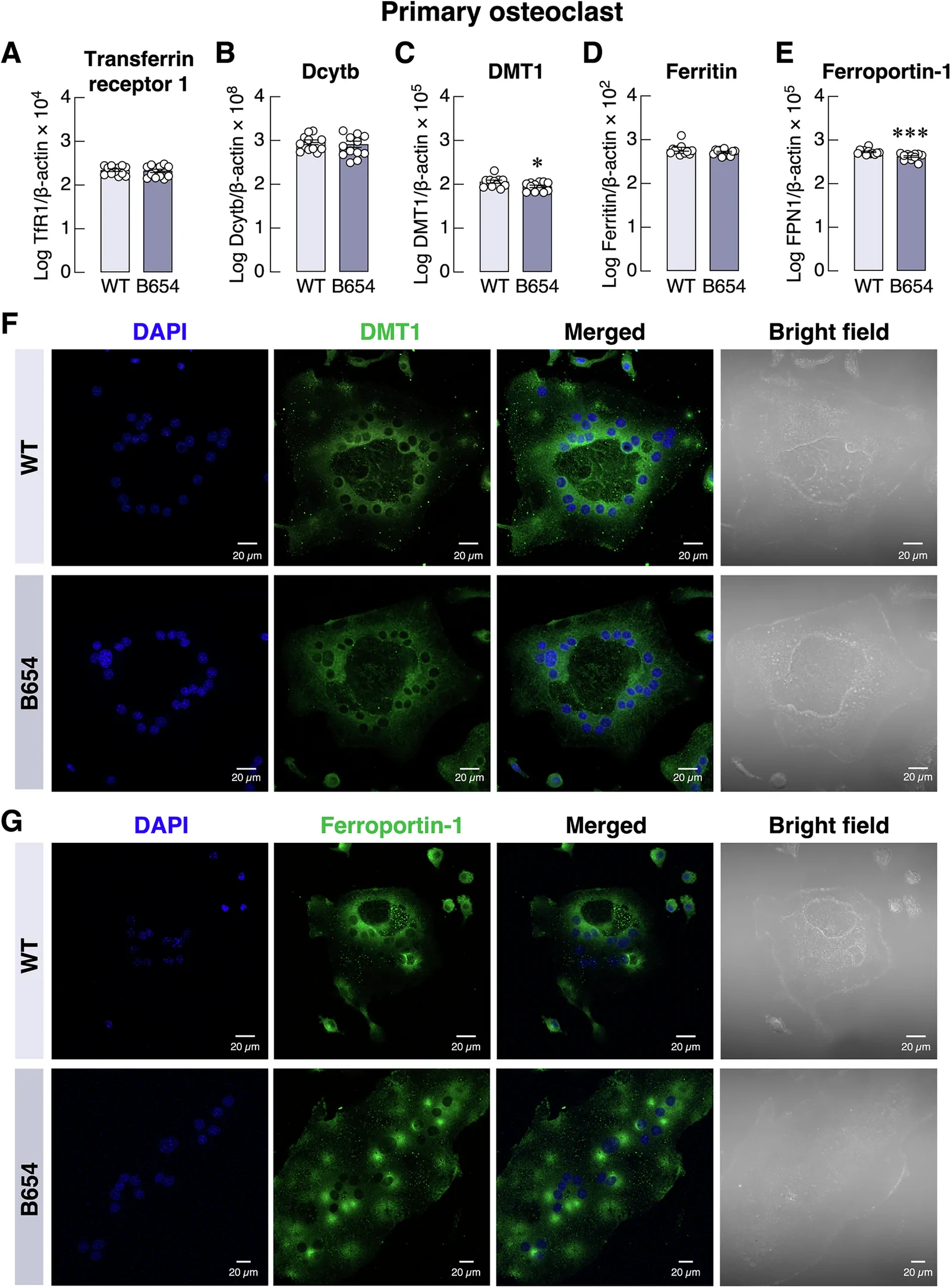
Downregulated mRNA, but not protein, expression of DMT1 and ferroportin-1 in primary osteoclasts derived from β-thalassemic mice. The mRNA expression of iron transporter genes, i.e., **A** transferrin receptor 1 (TfR1), **B** duodenal cytochrome b (Dcytb), **C** divalent metal transporter 1 (DMT1), **D** ferritin, **E** ferroportin-1 in primary osteoclasts. Data expressed as mean ± SE (*n* = 12). Representative **F** DMT1 and **G** ferroportin-1 protein expression and localization in primary osteoclasts derived from bone marrow cells of wild-type (WT) and hemizygous β^IVSII-654^ knock-in thalassemic (B654) mice which were captured by confocal microscrope. Scale bars, 20 μm (×40 magnification). *P < 0.05, ***P < 0.001 vs. age-matched WT by unpaired one-tailed Student’s *t* test.

Despite representative images and quantitative analyses showing fewer TRAP-positive multinucleated osteoclasts in B654 mice, the levels of medium CTX-1, a degradation product of bone resorption, were comparable to those in WT mice (Fig. 3A–C). Only RANK and Beclin 1 were upregulated in B654 mice (Fig. 3D–J). Furthermore, the levels of mRNA expression of some iron-transporting components, i.e., DMT1 and ferroportin-1, were significantly downregulated in B654-derived osteoclasts (Fig. 4). Immunofluorescence staining showed that both DMT1 and ferroportin-1 protein were predominantly localized in cytoplasm of multinucleated osteoclasts (Fig. 4). The expression level of ferroportin-1 transcript was negatively correlated with those of RANK and Beclin 1, but it was positively correlated with calcitonin receptor, cathepsin K, V-ATPase, and Nrf2 mRNA levels (Supplementary Fig. S1). Thus, ferroportin-1 may be involved in the regulation of osteoclastogenesis and osteoclast function.

### VIT-2763 had a modest effect on osteoblast-mediated bone formation

VIT-2763 was previously reported to bind with human ferroportin-1 [23]. Our molecular docking analysis showed that VIT-2763 could also bind to mouse ferroportin-1. VIT-2763 interacted with ferroportin-1 through multiple hydrogen bonds involving Asn185, Asp503, and Leu317. In addition, hydrophobic interactions, including van der Waals forces and π–π stacking between Tyr318 and the benzimidazole group of VIT-2763, further stabilized the binding complex (Supplementary Fig. S2A). Since enterocytes are known to abundantly express ferroportin-1, we used the Caco-2 cell line as a model of human intestinal epithelium-like cells. The Caco-2 cells formed a monolayer and were connected via tight junctions (Supplementary Fig. S2B). Here, VIT-2763 was found to downregulate ferroportin-1 protein expression not only in Caco-2 cells but also in the duodenum of B654 mice (Supplementary Fig. S2C, D). Cell viability of Caco-2 cells, but not UMR-106 and MLO-Y4 cells, was significantly increased by 20 μM VIT-2763 (Supplementary Fig. S2E–G).

**Fig. 5:**
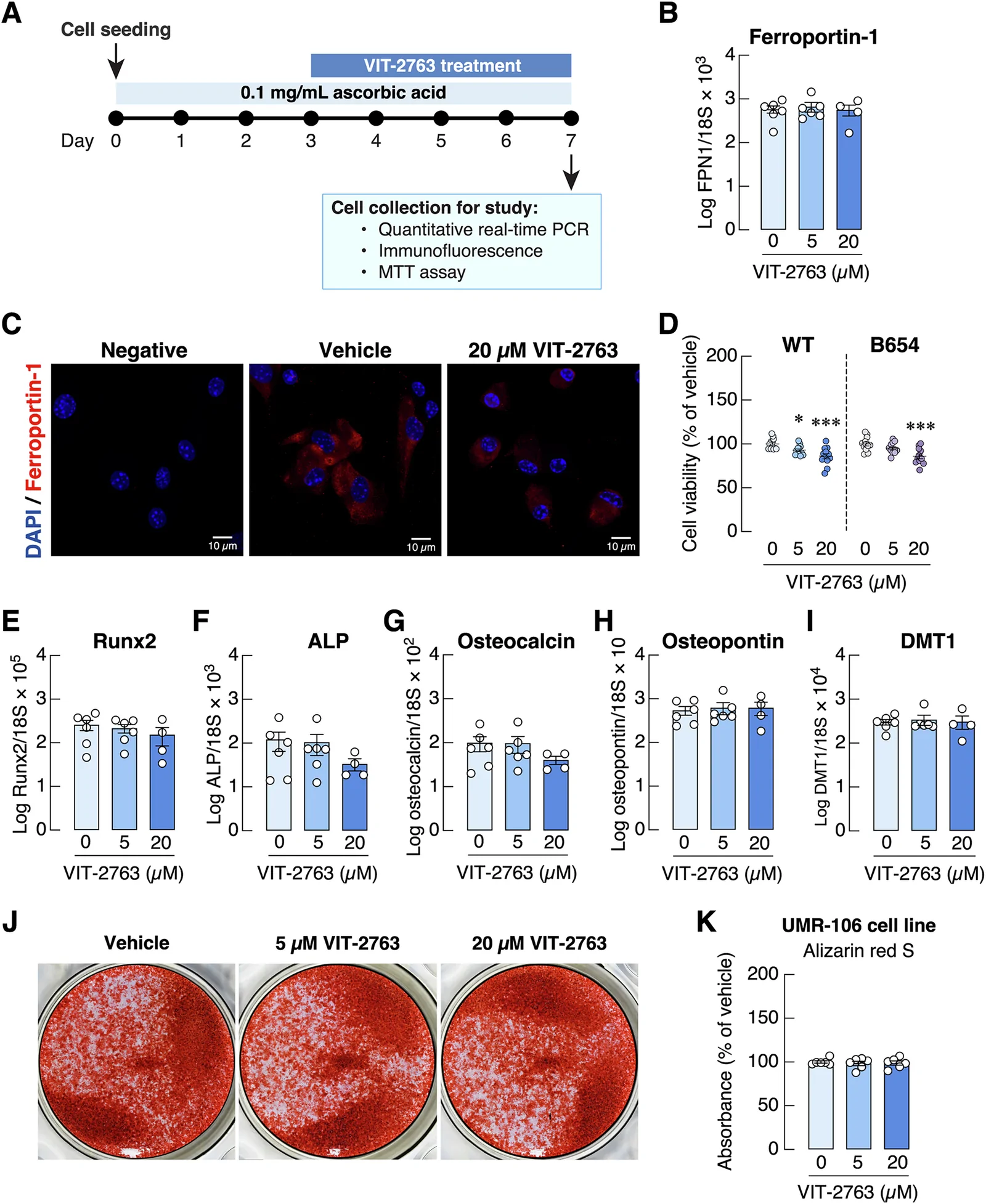
Modest effects of VIT-2763 on osteoblast-mediated bone formation. **A** A schematic picture shows experiment plan for primary osteoblast study. **B** mRNA and **C** protein expression of ferroportin-1 in primary osteoblasts of wild-type (WT) mice. Scale bars, 10 μm (×63 magnification). **D** Cell viability of primary osteoblasts from WT and hemizygous β^IVSII-654^ knock-in thalassemic (B654) mice after exposure to 5, 20 µM VIT-2763 for 4 days (*n* = 12). mRNA expression of osteoblast-specific genes, i.e., **E** Runx2, **F** alkaline phosphatase (ALP), **G** osteocalcin, **H** osteopontin, and iron transporter, i.e., **I** divalent metal transporter 1 (DMT1) in WT-derived primary osteoblasts after treatment with 5, 20 µM VIT-2763 for 4 days (*n* = 4–6). **J** Representative photomicrographs and **K** quantitative alizarin red staining of UMR-106 cell line after culture in media containing 8 mM β-glycerophosphate and 5, 20 µM VIT-2763 for 3 days (*n* = 6). Data expressed as mean ± SE. *P < 0.05, ***P < 0.001 vs. vehicle-treated group (0 μM) by one-way analysis of variance (ANOVA) with Dunnett’s multiple comparisons test.

Moreover, VIT-2763 did not alter mRNA expression but downregulated ferroportin-1 protein expression in primary osteoblasts (Fig. 5). Cell viability of primary osteoblast derived from WT and B654 mice was reduced approximately 14% after exposure to 20 μM VIT-2763 (Fig. 5). Additionally, mRNA expression of bone formation-related genes (i.e., Runx2, ALP, osteocalcin, and osteopontin), DMT1, and bone mineralization were not changed by VIT-2763 (Fig. 5). The transcripts related to the cell cycle (i.e., cyclin D1, Cdkn1a, and Cdkn2a) and apoptosis (i.e., Bcl-2, cytochrome c, caspase-9 and -3) were not altered by VIT-2763, although ferroportin-1 mRNA expression was positively correlated with most of those genes (Supplementary Fig. S3). Thus, VIT-2763 is likely to exert a modest effect on osteoblast-mediated bone formation.

### VIT-2763 attenuated osteoclastic bone resorption by delaying osteoclast differentiation but prolonging osteoclast survival

**Fig. 6:**
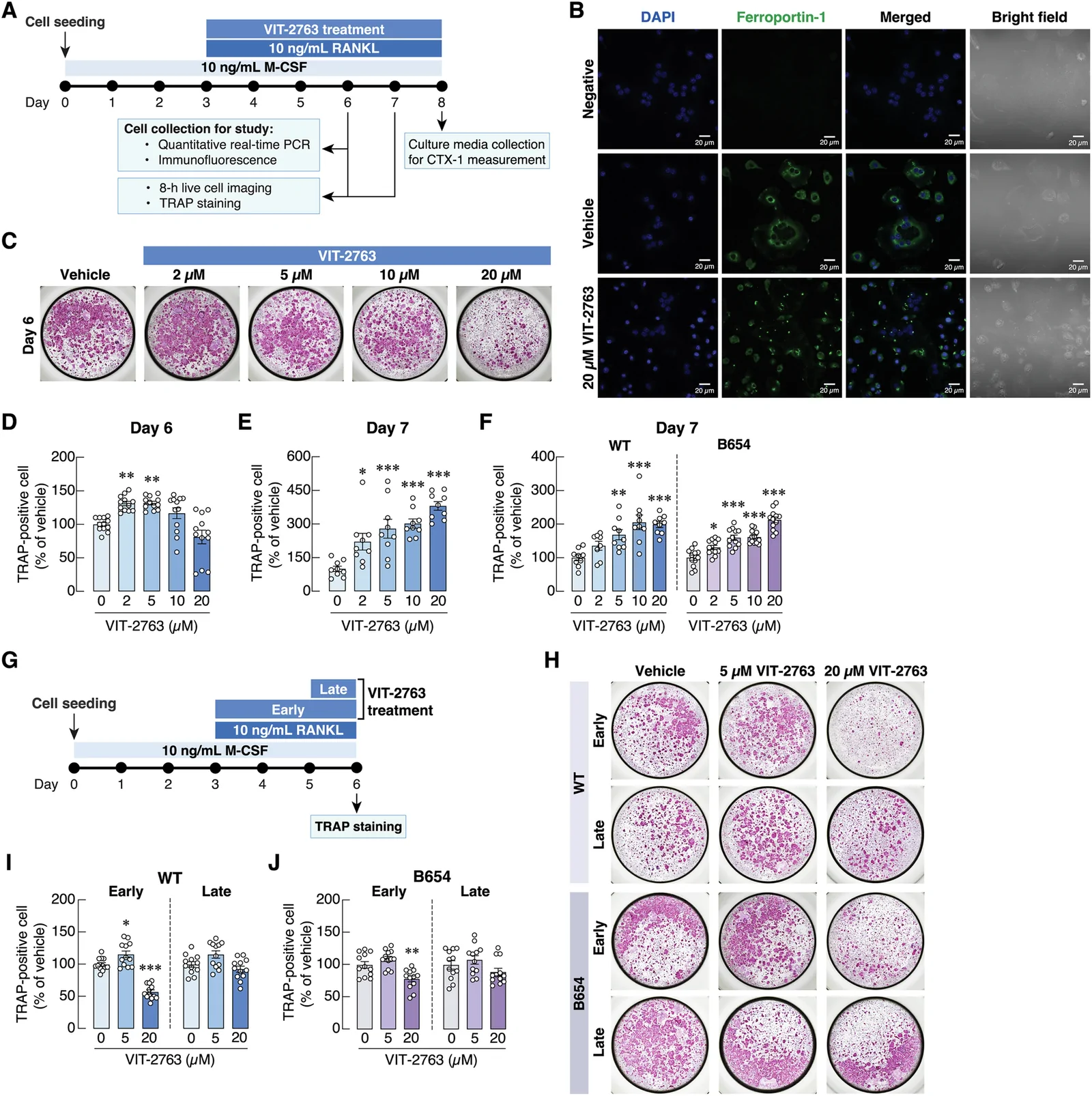
Delayed early osteoclast differentiation and prolonged osteoclast survival induced by VIT-2763 in vitro. **A** A schematic picture shows experiment timeline for primary osteoclast study. **B** Immunofluorescence staining of ferroportin-1 protein in primary osteoclasts derived from wild-type (WT) bone marrow cells after exposure to 20 µM VIT-2763 for 3 days which was captured by confocal microscope. Scale bars, 20 μm (×40 magnification). **C** Representative photomicrographs of TRAP-positive multinucleated osteoclasts from WT mice after culture for 6 days. Number of TRAP-positive cells were manually counted at **D** day 6 and **E**, **F** day 7 of culture after 2, 5, 10, 20 µM VIT-2763 treatment. **G** A schematic picture shows experiment timeline for VIT-2763 testing in primary osteoclasts during early and late osteoclast differentiation. **H** Representative TRAP staining and quantitative analysis of TRAP-positive multinucleated osteoclasts from **I** WT and **J** β^IVSII-654^ knock-in thalassemic (B654) mice after 5, 20 µM VIT-2763 treatment (*n* = 8–12). Data expressed as mean ± SE. *P < 0.05, **P < 0.01, ***P < 0.001 vs. vehicle-treated group (0 μM) by one-way analysis of variance (ANOVA) with Dunnett’s multiple comparisons test.

To investigate the effects of VIT-2763 on bone resorption, we cultured the bone marrow cells in media containing M-CSF and RANKL to induce pre-osteoclast differentiation (Fig. 6). The multinucleated osteoclasts were predominantly observed at day 6, then underwent apoptosis (Supplementary Video S1). The present study revealed that 20 μM VIT-2763 attenuated ferroportin-1 protein expression in primary osteoclasts (Fig. 6B). At day 6, the number of TRAP-positive multinucleated osteoclasts was increased by 2 and 5 μM VIT-2763, while it tended to decrease at 20 μM. TRAP-positive multinucleated osteoclasts were significantly increased after incubation with VIT-2763 until day 7 as observed in both WT and B654 mice (Fig. 6).

**Fig. 7:**
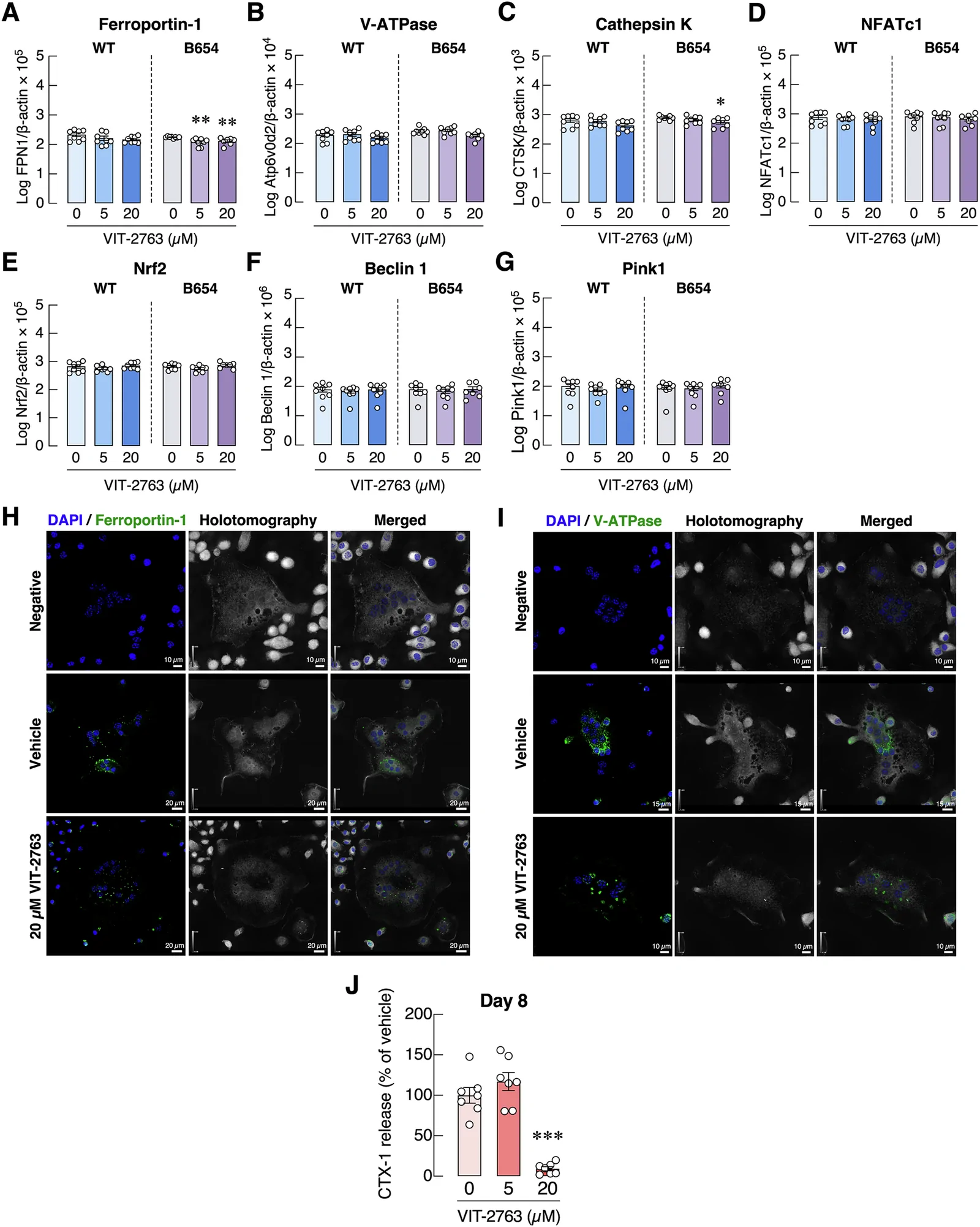
Attenuation of osteoclastic bone resorption by VIT-2763. The mRNA expression of **A** ferroportin-1, osteoclast-specific genes, i.e., **B** vacuolar ATPase H^+^ transporting V0 subunit D2 (ATP6v0d2 or V-ATPase), **C** cathepsin K (CTSK), signaling factor, i.e., **D** nuclear factor of activated T-cells c1 (NFATc1), transcription factor, i.e., **E** nuclear factor erythroid 2-related factor 2 (Nrf2), and mitophagy-related genes, i.e., **F** Beclin 1, **G** PTEN-induced kinase 1 (Pink1) in primary osteoclasts derived from bone marrow cells of wild-type (WT) and hemizygous β^IVSII-654^ knock-in thalassemic (B654) mice after treated with 5, 20 µM VIT-2763 for 3 days (*n* = 7–8). Representative three-dimensional (3D) hologram images with fluorescent labeling of **H** ferroportin-1 and **I** V-ATPase in WT-derived osteoclasts. Blue is DAPI stained nuclei. Green is either ferroportin-1 or V-ATPase protein. Scale bars, 10–20 μm (×40 magnification). **J** Concentration of C-terminal telopeptides of type I collagen (CTX-1) in culture media obtained from WT bone marrow-derived osteoclasts grown on bone slices treated with 5, 20 µM VIT-2763 (*n* = 7). Data expressed as mean ± SE. *P < 0.05, **P < 0.01, ***P < 0.001 vs. vehicle-treated group (0 μM) by one-way analysis of variance (ANOVA) with Dunnett’s multiple comparisons test.

To discriminate the effect of VIT-2763 on osteoclast differentiation, the osteoclast precursors were treated with VIT-2763, starting at day 3 or 5 of culture (Fig. 6G). The number of TRAP-positive osteoclasts was consistently increased in 5 μM VIT-2763. In contrast, 20 μM VIT-2763 effectively reduced early osteoclast differentiation, but not late differentiation, in both WT- and B654-derived osteoclasts (Fig. 6H–J). In WT-derived osteoclasts, VIT-2763 had no significant effects on the mRNA expression of ferroportin-1, osteoclast markers (V-ATPase and cathepsin K), signaling molecules (NFATc1 and Nrf2), and mitophagy-related genes (Fig. 7). However, VIT-2763 significantly attenuated mRNA expression of ferroportin-1 and cathepsin K in B654-derived osteoclasts. Furthermore, ferroportin-1 mRNA expression positively correlated with osteoclast- and mitophagy-related genes (Supplementary Fig. S4).

The representative 3D hologram images with fluorescent labeling revealed that ferroportin-1 and V-ATPase protein expression in primary osteoclasts were apparently downregulated by VIT-2763, a finding that was consistently observed in confocal images (Fig. 7 and Supplementary Fig. S5). A reduction in CTX-1 released from bone slices after treatment with 20 μM, but not 5 μM VIT-2763, supported the role of VIT-2763 in suppression of osteoclast-mediated bone resorption in vitro (Fig. 7).

### VIT-2763 biphasically regulated osteoclastogenesis in iron-loaded conditions

**Fig. 8:**
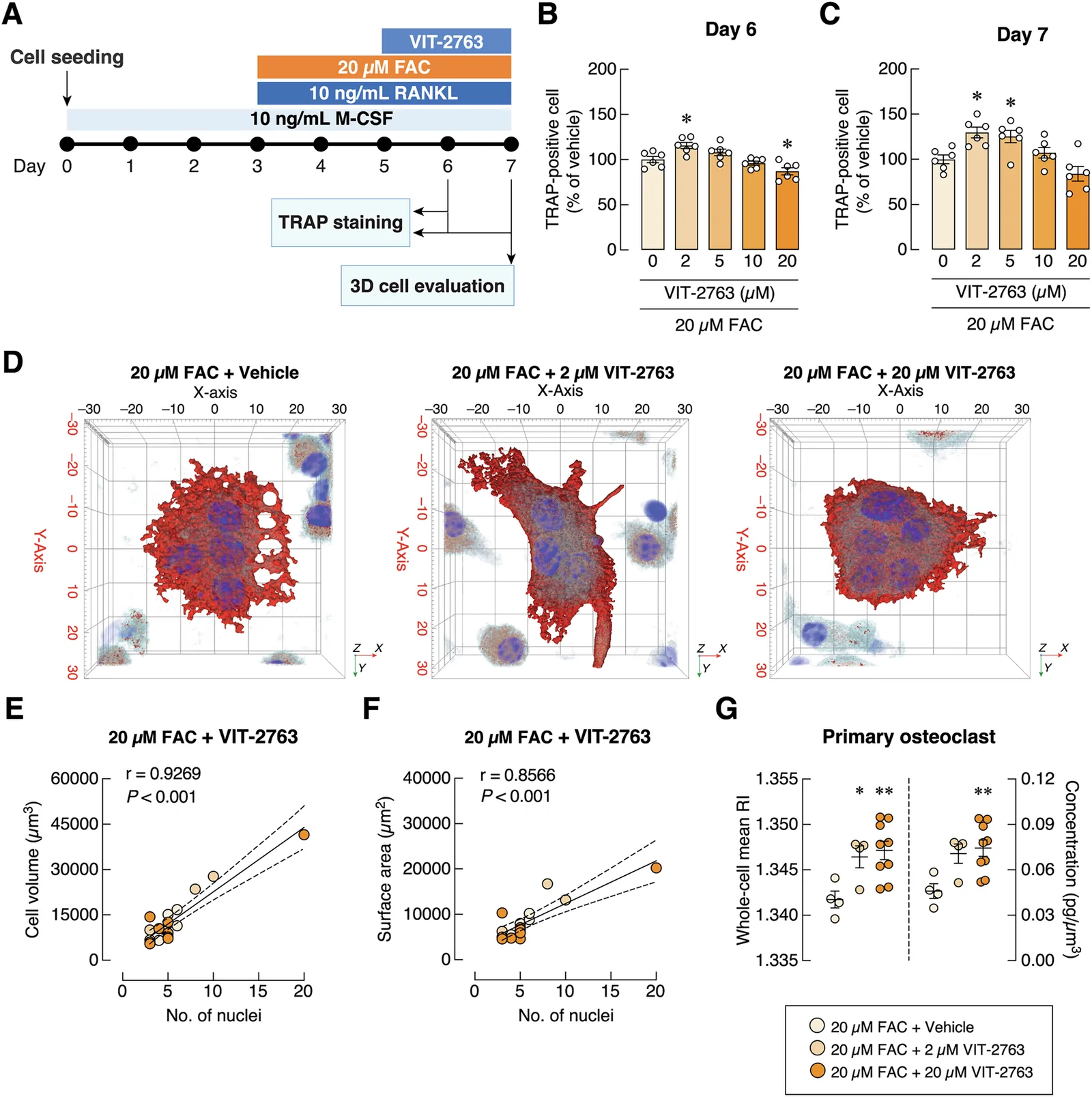
Biphasic regulation of osteoclastogenesis by VIT-2763 under iron-loaded conditions. **A** A schematic picture shows experiment timeline for primary osteoclast culture in media containing 20 µM ferric ammonium citrate (FAC). Number of TRAP-positive cells were manually counted at **B** day 6 and **C** day 7 after 2, 5, 10, 20 µM VIT-2763 treatment (*n* = 6). **D** The three-dimensional (3D) hologram images with DAPI staining were captured by holotomography microscope at day 7 of culture. Correlation plot between number of nuclei and **E** cell volume, **F** surface area in primary osteoclasts after 2, 20 µM VIT-2763 treatment (*n* = 4–9). Dashed line represents the 95% confident bands of the best-fit line. *r*, Pearson correlation coefficient. *P,* *p*-value of the correlation. **G** Whole-cell mean refractive index (RI) and concentration (dry mass/cell volume) were automatically evaluated by TomoStudio software version 3.3.9 (*n* = 4–9). Data expressed as mean ± SE. *P < 0.05, **P < 0.01 vs. vehicle-treated group (0 μM) by one-way analysis of variance (ANOVA) with Dunnett’s multiple comparisons test.

To elucidate the VIT-2763 action in iron-loaded conditions, we pre-treated the osteoclast precursors with 20 μM ferric ammonium citrate (FAC) prior to VIT-2763 treatment (Fig. 8A). The number of multinucleated osteoclasts increased with 2 μM VIT-2763 but was reduced with 20 μM VIT-2763 (Fig. 8). Unlike in normal media, TRAP-positive multinucleated osteoclasts did not increase with 10–20 μM VIT-2763 when the cells were cultured until day 7 (Fig. 8).

Representative 3D hologram images and quantitative analysis revealed that osteoclast morphology (i.e., volume, surface area, projected area, and sphericity) and property (i.e., dry mass) were unaltered by 2 or 20 μM VIT-2763 (Supplementary Table S3). Moreover, the number of nuclei was positively correlated with the volume and surface area of primary osteoclasts. Interestingly, the whole-cell mean RI and concentration were increased by VIT-2763 (Fig. 8). Thus, alterations of cellular properties by VIT-2763 may partly reduce osteoclastogenesis in iron-loaded conditions.

## Discussion

Cellular iron metabolism in osteoclasts profoundly affects their number and activity. Under normal conditions, ferroportin-1 contributes to iron efflux from osteoclast precursors and mature osteoclasts [10, 24] and likely helps prevent intracellular iron accumulation during iron-loading conditions. Our study demonstrated that B654 mice with iron overload exhibited bone microstructural defects and aberrant intestinal iron absorption. An in vitro study revealed that bone formation-related genes were upregulated in B654-derived osteoblasts, perhaps as a compensatory mechanism to counteract bone loss. The mRNA expression of osteoclast-specific genes was not altered, although the number of multinucleated osteoclasts was significantly decreased in B654 mice. Even though both osteoblasts and osteoclasts expressed iron-transporting machinery—e.g., transferrin receptor 1, DMT1, ferritin, and ferroportin-1—there have been few studies investigating how these components affect bone metabolism.

Ferroportin-1 was crucial for iron recycling after erythrophagocytosis by reticuloendothelial macrophages of liver, spleen, and bone marrow [25]. Ferroportin-1 mRNA expression declined during osteoblast maturation [18]. In osteoblasts, high intracellular iron concentration was reported to upregulate ferroportin-1 mRNA expression, while its expression was attenuated during low iron levels [26]. Furthermore, cellular iron deprivation also induced transferrin receptor expression [27, 28]. Downregulation of ferroportin-1 concurrently with upregulation of transferrin receptor 1 implied cellular iron demand in primary osteoblasts of B654 mice since iron was essential for pre-osteoblast proliferation [29]. Hence, a compensatory increase in iron entry and a decrease in iron export potentially brought intracellular iron concentration toward the physiological range, which, in turn, enhanced osteoblast proliferation and maturation in B654 mice. Consistently, ferroportin-1 mRNA expression was found to negatively correlate with bone formation-related genes in primary osteoblasts.

Our in vitro study showed that the ferroportin-1 mRNA levels in osteoclasts of B654 mice and the number of multinucleated osteoclasts were less than those of WT mice. Although ferroportin-1 mRNA expression was positively correlated with bone resorption-related genes, it did not reflect the resorptive activity of primary osteoclasts. The level of released CTX-1 from bone slice was comparable between WT- and B654-derived osteoclasts, suggesting a compensatory increase in resorptive activity despite a lower number of differentiated osteoclasts from B654 mice.

Previous studies demonstrated that ferroportin-1 mRNA expression fluctuated during early osteoclast differentiation and declined at late differentiation [18, 30]. Genetic deletion of ferroportin-1 expression in osteoclast precursors enhanced osteoclast differentiation and increased cellular iron accumulation in response to iron loading. However, loss of ferroportin-1 expression in late differentiated osteoclasts had no effect on multinucleated osteoclast formation and osteoclast function, even though an intracellular iron level was also elevated [24]. Therefore, in addition to iron exporter, ferroportin-1 plays some roles in osteoclast differentiation, particularly during the early phase of osteoclast formation.

Our study further showed that ferroportin-1 protein was abundantly expressed in the plasma membrane of mononuclear cells and in the cytoplasm of multinucleated osteoclasts. Since iron was essential for osteoclast proliferation and differentiation [24, 31], changes in cellular iron levels might determine the fate of osteoclast differentiation. We postulated that translocation of ferroportin-1 protein possibly regulated the cellular processes of osteoclasts. For instance, elevation of intracellular iron stimulated proliferation of osteoclast precursors without affecting cell survival [24]. Therefore, membrane localization of ferroportin-1 in the osteoclast precursors might facilitate iron efflux to prevent excess iron-induced cell death and hyperproliferation of osteoclast precursors, subsequently driving differentiation of osteoclast precursors. At a late stage, cytoplasmic localization of ferroportin-1 may increase cellular iron to supply heme proteins and iron-sulfur (Fe-S) clusters in mitochondria. Consequently, mitochondrial respiration and reactive oxygen species (ROS) production increased, which, in turn, promoted osteoclast maturation and function [32]. Thus, mature osteoclasts had higher levels of intracellular iron and ROS compared with bone marrow monocytes and osteoclast precursors [24, 31].

Besides enterocytes and hepatocytes, hepcidin also downregulated ferroportin-1 expression in osteoclasts. An increase in osteoclast number was thus a consequence of cytoplasmic iron accumulation following hepcidin treatment [4, 5, 17]. Since ferroportin-1 expression in the plasma membrane was partly upregulated by high cellular iron, hepcidin could overcome the effect of cellular iron overload by inducing ferroportin-1 internalization and degradation in macrophages, which were the osteoclast precursors [10]. Consequently, an increase in osteoclast number can be predicted in hepcidin treatment. Even though hepcidin treatment potentially alleviated iron overload-associated thalassemia [33, 34], it should be aware of the undesirable effects on bone.

The present study confirmed that ferroportin-1 protein was expressed by Caco-2 cell line, primary osteoblasts, and bone marrow cell-derived osteoclasts. The expression of ferroportin-1 protein was downregulated by VIT-2763. Notably, both mRNA and protein expression of ferroportin-1 were attenuated by VIT-2763, specifically observed in B654 osteoclasts. It is known that hepcidin is a master regulator of ferroportin-1 expression at the post-translational level. Although VIT-2763 was reported to mimic hepcidin action on ferroportin-1 expression [12], the translational regulation of ferroportin-1 by VIT-2763 has not been elucidated. The present study demonstrated that VIT-2763 could regulate ferroportin-1 expression at transcriptional levels in primary osteoclasts, particularly in B654-derived osteoclasts. Thereby, VIT-2763 might regulate ferroportin-1 expression in a cell-type-specific manner.

In primary osteoblasts, VIT-2763 reduced ferroportin-1 protein expression, but not mRNA expression, which was similar to hepcidin action [5, 16]. It was thus postulated that VIT-2763 modulated ferroportin-1 expression at post-transcriptional level in primary osteoblasts. Furthermore, VIT-2763 also decreased primary osteoblast viability even though there were no changes in cell cycle- and apoptosis-related markers. Primary culture usually contained a mixed population of osteoblasts, such as pre-osteoblasts, early differentiated osteoblasts, and mature osteoblasts. It is possible that the decrease in osteoblast viability caused by VIT-2763 partly resulted from ferroptosis induced by high cellular iron, particularly in early osteoblasts, which might be more sensitive to iron overload than more differentiated osteoblasts. Thus, cell viability of differentiated osteoblasts (i.e., UMR-106 cell line) and osteocytes (i.e., MLO-Y4 cell line) was not significantly altered by VIT-2763. Although VIT-2763 suppressed ferroportin-1 protein expression, it did not alter the expression of bone formation-related genes and bone mineralization. Taken together, once osteoblasts had fully differentiated into mature cells, VIT-2763 exerted insignificant effects on bone formation-related activities in vitro.

Regarding osteoclasts, osteoclasts are derived from hematopoietic stem cells, monocytes, or macrophages in bone marrow. In the presence of M-CSF and RANKL, osteoclast precursors differentiate into multinucleated osteoclasts and mature osteoclasts, respectively. The fully mature osteoclasts resorb bone and then undergo apoptosis [35, 36]. In our in vitro experiment, the multinucleated osteoclasts abundantly appeared at day 6 of culture. The number of TRAP-positive multinucleated osteoclasts rapidly decreased at day 7 of culture, suggesting an apoptosis of mature osteoclasts. The time-lapse imaging confirmed that fully differentiated osteoclasts underwent cell death as previously observed [37], specifically, the transition from day 6 to day 7 of culture when cells were grown on the Tomodish (Supplementary Video S1) as well as a plastic plate.

The present study demonstrated that low doses of VIT-2763 (2–5 μM) promoted the formation of multinucleated osteoclasts, while the osteoclast number tended to reduce after exposure to 20 μM VIT-2763. At day 7, the number of remaining osteoclasts was significantly higher after VIT-2763 treatment, presumably due to prolonged survival of osteoclasts. The results supported the functional role of VIT-2763 in delaying osteoclast differentiation rather than reducing osteoclast viability. It was unclear whether lower concentrations of VIT-2763 (2 and 5 μM) increased osteoclastogenesis. It was possible that an increase in intracellular iron concentration stimulated cell-cell fusion of osteoclast precursors during early osteoclast differentiation. Despite a greater number of multinucleated osteoclasts, the resorptive activity was unaltered in 5 μM VIT-2763 treatment as confirmed by a resorption pit assay. Interestingly, 20 μM VIT-2763 efficiently attenuated osteoclastogenesis and resorptive activity in vitro. We postulated that proper intracellular iron concentration coordinated with ferroportin-1 expression was essential to determine osteoclast differentiation and activity.

Additionally, an increase in TRAP-positive cells alone does not always contribute to bone loss (e.g., trabecular bone loss and cortical thinning). Elevation of resorptive activity, such as upregulation of V-ATPase and cathepsin K, formation of an actin ring at the sealing zone, also regulates osteoclast-mediated bone resorption. Acidification (i.e., H^+^ secretion via V-ATPase) and secretion of bone degrading-enzymes (i.e., TRAP, cathepsin K) at the resorption lacuna are essential for osteoclast-mediated bone dissolution. The present study revealed that mRNA expression of V-ATPase and cathepsin K was less changed, while V-ATPase protein expression was markedly suppressed by VIT-2763. Thus, VIT-2763 may regulate osteoclast-specific protein expression at the post-transcriptional level. Because osteoclast-mediated bone resorption is often inversely correlated with osteoclast survival, mature osteoclasts rapidly undergo apoptosis after resorption [38, 39]. We therefore postulated that VIT-2763 biphasically regulated osteoclastogenesis. Specifically, at high dose, VIT-2763 impaired osteoclast differentiation, maturation, and function, preventing full bone resorption. Consequently, resorptive activity decreased despite prolonged osteoclast survival.

Noticeably, most of our findings revealed that VIT-2763 consistently affected osteoclast-mediated bone resorption in vitro in both WT- and B654-derived osteoclasts. We postulated that VIT-2763 may potentially be used as an anti-resorptive agent for bone diseases, not limited only to β-thalassemic bone disease. However, VIT-2763 had some differential effects on the expression of some genes between WT and B654 osteoclasts. For instance, mRNA expression of ferroportin-1 and cathepsin K was suppressed by VIT-2763 only in B654 but not in WT osteoclasts. Thus, genotypic sensitivity to the drug was observed in primary osteoclasts derived from WT and B654 mice. Further in vivo study will be needed to confirm the genotypic difference of VIT-2763 on bone cells or bone microstructure.

In iron-loaded conditions, a high-dose VIT-2763 apparently reduced multinucleated osteoclast formation but did not prolong osteoclast survival. Our studies revealed that morphological properties of osteoclasts (i.e., volume, surface area, and projected area) were not significantly altered by VIT-2763 as evaluated by RI-based 3D holotomography. Slight increases in the whole-cell mean RI of primary osteoclasts after VIT-2763 treatment were suggested to be associated with increases in the concentration of intracellular molecules, which subsequently attenuated osteoclastogenesis and osteoclast activity.

Generally, ATP is essential for cellular processes, and its utilization increases during osteoclast differentiation [40]. Mature osteoclasts often contain more mitochondria than osteoclast precursors. Mitochondrial dysfunction could result in cellular ATP depletion, which, in turn, accelerates osteoclast-mediated bone resorption and leads to apoptosis [39]. Regulation of mitochondrial function and activity is important to control bone metabolism. The balance between mitochondrial autophagy—also known as mitophagy—and biogenesis is important to maintain energy supply to the cells. For instance, mitophagy of red blood cells (RBCs) was crucial for a shift in energy production during maturation. Impaired RBC mitophagy resulted in increased ROS levels and RBC apoptosis, particularly in β-thalassemia [13, 41]. Aoki et al. demonstrated that autophagy was increased during osteoclast differentiation, especially in very active osteoclasts [37]. ROS was also produced by mitochondrial respiration [40], and activation of RANKL-RANK signaling also increased intracellular ROS levels during osteoclast differentiation [42]. Enhanced autophagy likely prevented ROS-induced cellular damage, thereby promoting osteoclast differentiation. In contrast, mitophagy was progressively reduced in terminally differentiated osteoclasts, resulting in cellular ROS accumulation and induction of osteoclast apoptosis [37, 40]. Interestingly, VIT-2763 effectively alleviated ineffective erythropoiesis and anemia in β-thalassemic mice by inducing mitophagy in RBCs during cellular maturation. A reduction in ROS production after VIT-2763 treatment may help prolong RBC lifespan in β-thalassemic mice [12, 14]. Collectively, we postulate that VIT-2763 potentially regulates mitochondrial activity, ROS production, and cellular properties, which, in turn, mitigate osteoclast differentiation and bone resorption.

The present study was limited by the absence of direct measurements of intracellular iron and ROS levels, as well as mitochondrial function in osteoclasts. However, previous studies consistently revealed that attenuation of ferroportin-1 expression by genetic manipulation [24], hepcidin [43], or VIT-2763 treatment [12] caused an increase in intracellular iron levels in various cell types, such as osteoclasts, erythroblasts, and macrophages. Activation of the Nrf2-ferroportin-1 pathway reduced cellular iron, inhibited osteoclastogenesis, and ameliorated ovariectomy (OVX)-induced bone loss [44]. Since Beclin 1 and Pink1 are mitophagy-related factors, their mRNA and protein expression were increased during osteoclast differentiation [40]. Our study demonstrated that expression of Nrf2 and mitophagy-related genes was not changed by VIT-2763 treatment. Thereby, we postulated that VIT-2763 delayed osteoclast differentiation and suppressed bone resorption in Nrf2-, Pink1-, and Beclin 1-independent mechanisms. Nevertheless, further study will be needed to confirm the levels of intracellular iron, ROS, and mitochondrial function in osteoclasts during VIT-2763 treatment.

Although VIT-2763 was reported to mimic hepcidin action by inducing ferroportin-1 internalization and degradation, it also had additional benefits beyond ferroportin-1 suppression in the condition of β-thalassemia. For instance, VIT-2763 could improve ineffective erythropoiesis by decreasing the formation of insoluble α-globin aggregates and modulating mitophagy of RBCs [12, 13]. In bone cells, decreased ferroportin-1 expression by genetic modification or hepcidin treatment increased intracellular iron and accelerated osteoclast differentiation [17, 24]. On the other hand, the present study demonstrated that downregulation of ferroportin-1 by VIT-2763 delayed osteoclast differentiation and attenuated osteoclast-mediated bone resorption but only had minimal effects on osteoblasts. In high-iron condition, holotomographic analysis revealed that cellular properties, such as whole-cell RI, dry mass/cell volume, were also altered by VIT-2763 treatment. In addition to regulated iron export via ferroportin-1, VIT-2763 might have other roles in bone metabolism.

Besides direct action on bone cells, VIT-2763 possibly has indirect action on bone by alleviating iron overload. Our pilot study found that VIT-2763 effectively attenuated ferroportin-1 protein expression in the duodenum of B654 mice. Since intestinal iron absorption was negatively correlated with intestinal calcium absorption [9], we postulated that VIT-2763 may indirectly alleviate thalassemic bone resorption by regulating whole-body mineral homeostasis, at least by decreasing intestinal iron absorption. In future studies, investigation of bone microstructure and mineral homeostasis (e.g., calcium and iron) following in vivo VIT-2763 treatment in β-thalassemic mice may help confirm its potential benefits for thalassemic bone disease, as genotype-dependent sensitivity to the drug was observed in vitro.

In conclusion, our study demonstrated the potential benefits of the ferroportin-1 inhibitor VIT-2763, which is suitable for oral administration, in bone cell function. Downregulation of ferroportin-1 expression by VIT-2763 had only modest effects on osteoblast-mediated mineralization and perhaps bone formation. Conversely, VIT-2763 preferentially inhibited early osteoclast differentiation and osteoclast-mediated bone resorption, at least in part by altering cellular properties. This finding highlights the potential of VIT-2763 as a novel anti-resorptive agent under thalassemic conditions. Therefore, modulation of ferroportin-1 expression and cellular functions by VIT-2763 may represent a promising strategy for treating thalassemic osteopathy.

## Materials and methods

Expanded materials and methods are provided in the Supplementary Materials and Methods section and Supplementary Table S1.

### Animals

Female hemizygous β^IVSII-654^ knock-in thalassemic (B654) and age-matched wild-type (WT) C57BL/6 mice (8–20 weeks old; weighing 18–30 g) obtained from the Thalassemia Research Center, Institute of Molecular Biosciences, Mahidol University, were used. The B654 mice exhibited β-thalassemia intermedia as previously described [19, 20]. All experimental protocols were approved by the Institutional Animal Care and Use Committee (IACUC) of the Faculty of Science, Mahidol University (Protocol No. MUSC66-042-672). Our study was performed in accordance with relevant guidelines, e.g., ARRIVE guidelines (www.ARRIVEguidelines.org) and AVMA Guidelines for the Euthanasia of Animals (2020 Edition).

### Experimental design

After euthanasia, bones, intestine, and blood were collected from 16-week-old WT and B654 mice to determine complete blood count (CBC), tibial bone microstructure, mRNA expression of duodenal iron transporter genes, serum ferritin and transferrin by automated hematology analyzer, ultra‑high resolution micro-computed tomography (μCT), quantitative real-time PCR, and enzyme-linked immunosorbent assay (ELISA), respectively.

Primary osteoblasts were isolated from tibiae and femora of WT and B654 mice. After confluent, the primary osteoblasts were plated for studying cell viability, mRNA expression of osteoblast-related genes, and ferroportin-1 protein expression and localization under basal condition as well as VIT-2763 (catalog no. HY-112220; MedChemExpress, NJ, USA) treatment. UMR-106 and MLO-Y4 cell lines were used as models of differentiated osteoblasts and osteocytes, respectively.

For primary osteoclasts, bone marrow cells from tibiae and femora were isolated and cultured in media containing 10 ng/mL macrophage-colony stimulating factor (M-CSF) (catalog no. 216-MC-025; R&D Systems, MN, USA), 10 ng/mL receptor activator of nuclear factor-κB ligand (RANKL) (catalog no. 462-TEC-010; R&D Systems, MN, USA), with or without VIT-2763. The primary osteoclasts were collected for evaluation of tartrate-resistant acid phosphatase (TRAP) staining, mRNA expression of osteoclast-related genes, expression and localization of the specific proteins (i.e., ferroportin-1 and V-ATPase), and 3D cellular morphology and properties.

### Three-dimensional (3D) holotomographic imaging

The 3D holograms with fluorescence labeling of Caco-2 cells and primary osteoclasts were captured by a holotomographic microscope equipped with a 3D fluorescence imaging system (Tomocube, Inc.). The holographic images were reconstructed based on refractive index (RI). Time-lapse video of label-free live cell imaging of primary osteoclasts was constructed by continuously capturing hologram images every 20 min for 8 h during day 6 to 7 of culture. Quantitative analysis of cellular morphology and properties was automatically evaluated by TomoStudio software.

### Statistical analysis

Sample sizes were determined based on a power calculation providing an 80% probability of detecting a significant difference (*P* < 0.05). Individual data points are presented as dots in each graph. The *n* values are provided in the figure legends and represent the number of animals per group in in vivo experiments or the number of independent replicates in in vitro experiments. Key parameters, such as bone volume fraction (BV/TV), cell viability, and CTX-1 release, were assessed for normality and homogeneity of variance. As no significant deviation from these assumptions was detected, parametric statistical tests were applied. Results are presented as mean ± standard error (SE). Comparisons between two groups were performed using an unpaired one-tailed Student’s *t* test. One-way analysis of variance (ANOVA) followed by Dunnett’s multiple comparisons test was used for comparisons among multiple independent groups. Statistical significance was defined as *P* < 0.05. All analyses were performed using GraphPad Prism 9 (GraphPad Software, San Diego, CA, USA).

## Supplementary information

Supplementary Materials and Methods

Supplementary Tables S1–S3

Supplementary Figures S1–S5

Supplementary Video S1

Supplementary Video Legend

## Data Availability

The data supporting this study’s findings are available from the corresponding author upon reasonable request.
