# Supplementary material for: VIT-2763, a ferroportin-1 inhibitor, modulates osteoblast and osteoclast functions in hemizygous βIVSII-654 knock-in thalassemic mice with osteopenia
Source: Cell Death Discov. 2026 Jul 31;12:429. doi: 10.1038/s41420-026-03273-x (PMC13714295; doi:10.1038/s41420-026-03273-x)
Supplement: Supplementary file 1 [file 41420_2026_3273_MOESM1_ESM.pdf]

Supplementary Materials and Methods

ORIGINAL ARTICLE

VIT-2763, a ferroportin-1 inhibitor, modulates osteoblast and osteoclast functions in hemizygous $\beta^{IVSII-654}$ knock-in thalassemic mice with osteopenia

Ratchaneevan Aeimlapa[1,2], Jarinthorn Teerapornpuntakit[1,3], Jirawan Thongbunchoo[1,2], Pathnaree Wattano[1], Jiraporn Panmanee[4], Saovaros Svasti[5], Narattaphol Charoenphandhu[1,2,6,7,*]

[1] Center of Calcium and Bone Research (COCAB), Faculty of Science, Mahidol University, Bangkok, Thailand
[2] Department of Physiology, Faculty of Science, Mahidol University, Bangkok, Thailand
[3] Physiology Division, Preclinical Science, Faculty of Medicine, Thammasat University, Pathum Thani, Thailand
[4] Research Center for Neuroscience, Institute of Molecular Biosciences, Mahidol University, Nakhon Pathom, Thailand
[5] Thalassemia Research Center, Institute of Molecular Biosciences, Mahidol University, Nakhon Pathom, Thailand
[6] Institute of Molecular Biosciences, Mahidol University, Nakhon Pathom, Thailand
[7] The Academy of Science, The Royal Society of Thailand, Bangkok, Thailand

Running title: Anti-resorptive effects of ferroportin-1 inhibitor

*** Corresponding author**

Narattaphol Charoenphandhu, M.D., Ph.D.
Department of Physiology
Faculty of Science, Mahidol University
Rama VI Road, Bangkok 10400
Thailand
E-mail: naratt@narattsys.com

Keywords: β-thalassemia; vamifeport (VIT-2763); three-dimensional (3D) holotomography; osteoblast; osteoclast

Animals

Female hemizygous $\beta^{IVSII-654}$ knock-in thalassemic (B654) and age-matched wild-type (WT) C57BL/6 (8–20 weeks old; weighing 18–30 g) were housed in polystyrene cages under a 12:12-hour light-dark cycle, at a room temperature of 21–23 °C, and a humidity of 50–60%. They were fed standard laboratory chow containing 1.0% calcium and 0.9% phosphorus (C.P. Company, Thailand) and given RO water ad libitum in an Association for Assessment and Accreditation of Laboratory Animal Care International (AAALAC)-accredited facility. All experimental protocols have been approved by the Institutional Animal Care and Use Committee (IACUC) of the Faculty of Science, Mahidol University. Our study was performed in accordance with relevant guidelines and regulations, including the ARRIVE guidelines (www.ARRIVEguidelines.org). Mice were randomly allocated to experimental groups based on genotype (WT or B654). Therefore, no additional randomization procedures were applied.

Anesthesia and euthanasia

For complete blood count (CBC) measurement, mice were anesthetized with 2–5% isoflurane in an induction chamber. Following blood collection, the mice were returned to their home cage after hemostasis was achieved, and full recovery from anesthesia was confirmed.

For the other experiments, mice were euthanized by intraperitoneal injection of xylazine (15 mg/kg i.p.; L.B.S. Laboratory, Bangkok, Thailand) and tiletamine/zolazepam (120 mg/kg i.p.; Virbac Laboratories, Carros, France). Tissues, blood, or cells were collected for analyses. Cardiac excision was performed to ensure humane euthanasia.

Complete blood count (CBC)

After anesthesia, whole blood was collected from the submandibular vein by puncture with a 25-gauge needle and then transferred into an ethylenediaminetetraacetic acid (EDTA)-coated tube. The CBC was automatically measured by VETSCAN® HM5 Hematology Analyzer.

Measurement of serum ferritin and transferrin

The levels of serum ferritin (catalog no. 80636; Crystal Chem, IL, USA) and transferrin (catalog no. 80661; Crystal Chem, IL, USA) were determined by commercial ELISA kits according to the manufacturers' protocols.

Ultra-high resolution micro-computed tomography (µCT)

After removal of the attached soft tissues, the tibiae were wrapped in moist gauze to prevent dehydration. An *ex vivo* scan was performed using ultra focus mode at 50 kV, 0.21 mA (E-Class VECTor6 CT system, MILabs, Netherlands). The rotation angle was 0.25° at each step and voxel size was 5 μm^3 isotropically. Three-dimensional figures were reconstructed by MILabs Rec 12.00 software. Microstructural parameters of trabecular region, i.e., bone volume fraction (BV/TV, %), trabecular thickness (Tb.Th, µm), trabecular separation (Tb.Sp, µm), connectivity density (Conn.D, mm^{-3}), were analyzed at 0.3–1 mm distal to the proximal growth plate. Cross-sectional area (CSA, mm^2), cortical thickness (Ct.Th, mm), and periosteal perimeter (Ps.Pm, mm) were analyzed at tibial midshaft. All parameters were analyzed by BoneJ [ImageJ plugin version 1.50e, National Institutes of Health (NIH)].

Primary osteoblast culture

Primary osteoblasts were prepared from the mouse tibiae and femora of 12–20 weeks old female mice and maintained as previously described by Charoenphandhu et al. [1].

Primary osteoclast culture

Primary osteoclasts were derived from the bone marrow cells of long bones from 9–12 weeks old female mice. The differentiation of osteoclast precursors was induced by supplementing with 10 ng/mL macrophage colony-stimulating factor (M-CSF) and 10 ng/mL receptor activator of nuclear factor-κB ligand (RANKL) according to Charoenphandhu et al. [1]. For the iron-loaded experiment, the cells were maintained in α-media containing 20 μM ferric ammonium citrate (FAC) (catalog no. F5879; Sigma) prior to VIT-2763 treatment. The multinucleated osteoclasts were manually counted on day 6–7 of culture.

Cell viability test

The Caco-2 [American Type Culture Collection (ATCC) no. HTB-37], UMR-106 [American Type Culture Collection (ATCC) no. CRL-1661], MLO-Y4 [Accession ID. CVCL_M098; catalog no. EKC002; Kerafast] cells were plated at 2×10^4, 1×10^3, 3×10^3 cells/well, respectively, in a 96-well plate. After 24 h, the cells were incubated with media containing various concentrations of VIT-2763 (i.e., 0, 2, 5, 10, 20 μM). Cell viability was tested at 24, 48, and 72 h after exposure to VIT-2763. For primary osteoblasts, the confluent osteoblasts were seeded in a 96-well plate at 5×10^4 cells/well. After 72 h, the cells were incubated with media containing 5 or 20 μM VIT-2763 for 4 days. The cells were then added with 20 μL 3-(4,5-dimethylthiazol-2-yl)-2,5-diphenyltetrazolium bromide (MTT) reagent (5 mg/mL; catalog no.

M2128; Sigma) and incubated at 37 °C. After 3 h, the culture media were removed, and the cells were incubated with 100 µL of DMSO at 37 °C for 5–10 minutes. Absorbance was measured at 540 nm by a microplate reader (model 1420; Wallac, Turku, Finland). Cell viability was expressed as a percentage of the vehicle (0 µM) by converting the absorbance of the vehicle wells to 100%.

Quantitative alizarin red S staining

Alizarin red S staining was modified from Stanford et al. [2]. UMR-106 cell line was plated at 2.5×10^4 cells/well in 12-well plates and cultured in Dulbecco's modified Eagle's medium (DMEM; Sigma, St. Louis, MO, USA) supplemented with 10% fetal bovine serum (FBS; PAA Laboratories, Pasching, Austria), and 100 U/mL penicillin–streptomycin (Gibco, Grand Island, NY, USA). Cells were incubated at 37 °C with 5% CO_2 for 72 h. The cells then were cultured in DMEM containing 8 mM β-glycerophosphate (catalog no. G9422; Sigma) with or without VIT-2763 for 72 h. After washing with 1× PBS, the cells were fixed with ice-cold 70% ethanol for 30 min then rinsed with distilled water. The cells were stained with 40 mM alizarin red (pH 4.2) at room temperature for 10 min followed by rinsed with distilled water. Quantitative alizarin red S staining was performed by dissolved with 10% w/v cetylpyridinium chloride (CPC) (pH 7.0) for 15 min at room temperature. The 5-fold dilution of alizarin red extracts was measured at 562 nm by a multimode microplate reader (model Spark 10M; TECAN, Männedorf, Switzerland). The absorbance was expressed as a percentage of the vehicle (0 µM) by converting the absorbance of the vehicle wells to 100%.

Tartrate-resistant acid phosphatase (TRAP) staining

The bone marrow cells were plated at 1×10^5 cells/well in 96-well plates and cultured in α-MEM containing osteoclastogenic factors. The cells were treated with 5 or 20 μM VIT-2763 from days 3 to 7. The cells were fixed and stained by using TRAP staining kit (catalog no. PMC-AK04F-COS; Cosmo Bio, Tokyo, Japan). TRAP-positive multinucleated osteoclasts with ≥3 nuclei were visualized and manually counted as previously described [3, 4].

Quantitative real-time PCR

Primary osteoblasts were plated at 3×10^5 cells/well and 1.5×10^5 cells/well in 6-well and 12-well plates, respectively. The bone marrow cells were plated at 5×10^5 cells/well in 6-well plates. After 3 days of plating, the cells were treated with 5 or 20 μM VIT-2763. On day 6 or 7, total RNA was extracted using TRIzol reagent (Invitrogen, Carlsbad, CA, USA) as described by Charoenphandhu et al. [1]. The exclusion criterion for the real-time PCR experiments was an insufficient total RNA concentration for cDNA synthesis; therefore, samples that did not meet this criterion were excluded from downstream analysis. The primers used in this study were listed in Supplementary Table S1. Changes in gene expression were calculated from the threshold cycles (C_t).

Immunofluorescence

The bone marrow cells were plated at 5×10^5 cells/well in TomoDish (Tomocube, Inc., Daejeon, Republic of Korea) and cultured in α-MEM containing 10 ng/mL M-CSF and 10 ng/mL RANKL for 6 days. For Caco-2 cells, cells were seeded at 4×10^5 cells/well in 8-well cell culture slide (SPL Life Sciences, Gyeonggi-do, South Korea) 3 days prior to VIT-2763 treatment

for 18 h. Cells were fixed with 4% paraformaldehyde at room temperature for 20 min. Nonspecific binding was blocked with 5% normal goat serum (catalog no. 005-000-121; Jackson Immuno Research, PA, USA) or 5% normal donkey serum (catalog no. 017-000-121; Jackson Immuno Research, PA, USA) in phosphate-buffered saline (PBS). Cells were incubated with rabbit polyclonal primary antibody against ferroportin-1 (1:100; catalog no. PA5-22993; Invitrogen, Thermo Scientific, MA, USA), DMT1 (1:100; catalog no. 20507-1-AP; Proteintech), or V-ATPase (1:100; catalog no. SAB2103221; Sigma) at 4 °C overnight. The primary antibody was omitted for negative control. Each interested protein in primary osteoclasts was detected by incubating with goat anti-rabbit IgG conjugated with fluorescein (FITC) (1:100; catalog no. 111-095-003; Jackson Immuno Research) for 1 h in dark condition, whereas donkey anti-rabbit IgG conjugated with Alexa 594 (1:200; catalog no. 711-585-152; Jackson Immuno Research) was used to detect ferroportin-1 expression in Caco-2 cells. Cells were then stained with 4′,6-diamidino-2-phenylindole (DAPI). The fluorescent signals were visualized and captured by confocal laser-scanning microscope (model Zeiss LSM800; Carl Zeiss AG, Germany).

Three-dimensional (3D) holotomographic imaging

Caco-2 cells were seeded at 1×10^5 cells/well in TomoDish then culture for 5 days. Label-free Caco-2 monolayer was captured under a 40× objective lens with a numerical aperture of 0.95 air using a holotomographic microscope equipped with 3D fluorescence imaging system (model HT-X1; Tomocube, Inc.). The 3D holographic images were acquired by TomoStudio X version 1.6.9 and generated by TomoAnalysis version 1.8.3b software based on refractive index (RI) range around 1.339–1.361 and 1.320–1.350 which defined as whole cell and tight junction, respectively.

For primary osteoclasts, bone marrow cells were plated at 5×10^5 cells/well in TomoDish and cultured in α-MEM containing osteoclastogenic factors for 6–7 days. During transition from day 6 to 7, time-lapse holotomograms were continuously captured every 20 min for 8 h. Time-lapse video of label-free live cell imaging of primary osteoclasts was constructed. Immunostaining of interested proteins (i.e., ferroportin-1 and V-ATPase) was performed for studying expression and localization. The fluorescence signals were visualized and captured under a 40× objective lens with a numerical aperture of 0.95 air using a HT-X1 holotomographic microscope equipped with 3D fluorescence imaging system (Tomocube, Inc.) with TomoStudio X version 1.6.9. The 3D hologram images were reconstructed based on RI distribution of each cellular compartment using TomoStudio X version 1.6.9 and TomoAnalysis version 1.8.3b software.

For quantitative analysis of cellular morphology and properties, cells were fixed with 4% paraformaldehyde at room temperature for 20 min. After that, the nuclei were stained with DAPI. Cells with 3 or more nuclei were identified as primary osteoclasts. The primary osteoclasts were captured under a 60× objective lens with a numerical aperture of 1.2 (water immersion) using a holotomographic microscope with 3D fluorescence imaging system (model HT-2H; Tomocube, Inc.). The 3D hologram images were then reconstructed. Morphology and properties of primary osteoclasts were automatically evaluated by TomoStudio version 3.3.9. For tile-scan datasets, the large field image was generated by stitching individual tiles by TomoStitcher version 1.2.0 software. The exclusion criterion for 3D hologram image analysis was the presence of additional cells detected within the ROI by the analysis software.

Bone resorption pit assay

Bone marrow cells were plated at a density of 1 × 10^5 cells/well on bone slices (catalog no. DT-1BON1000-96; Immunodiagnostic Systems, Tyne and Wear, UK) in 96-well plates and then cultured in α-MEM containing osteoclastogenic factors for 8–13 days. On day 3, the osteoclast precursors growing on the bone slices were incubated with 5 or 20 μM VIT-2763 until day 8. The culture media were collected to determine the levels of released C-terminal telopeptides of type 1 collagen (CTX-1) in the supernatant using a crosslaps for cell culture ELISA kit (catalog no. AC-07F1; Immunodiagnostic Systems).

Flame atomic absorption spectroscopy (FAAS)

One mg of femoral ash was digested with a mixture of 65% HNO_3 and 37% HCl (8:1 ratio, v/v). The digested samples were then diluted with deionized water to achieve a final volume of 50 mL. Iron concentration was measured by a flame atomic absorption spectrophotometer (model PinAAcle 900T; PerkinElmer). Femoral iron was calculated and expressed as the weight of femoral iron per femoral ash weight.

***In vivo* VIT-2763 treatment, tissue preparation, immunostaining, and tissue imaging**

After acclimatization, 8-week-old female mice were divided into 3 groups: WT-Vehicle, B654-Vehicle, and B654-VIT. B654 mice were orally administered 30 mg/kg VIT-2763 daily for 3 days prior to sacrifice. The isolated duodenum was washed with 1× PBS, then fixed in 4% w/v paraformaldehyde at 4 °C overnight. The duodenum was dehydrated in graded ethanol, then cleared in xylene. After being embedded in paraffin, a specimen was cut longitudinally with a microtome (model RM2255; Leica, Nussloch, Germany) in 4-μm-thickness. The sections were

deparaffinized in xylene and rehydrated in graded ethanol. The antigen retrieval was performed by incubating the duodenal sections in Tris/EDTA buffer (pH 9.0) at 95 °C for 30 min. Nonspecific binding was blocked by blocking solution containing 4% bovine serum albumin (BSA) and 5% normal goat serum (catalog no. 005-000-121; Jackson Immuno Research, PA, USA) in 1× PBST at room temperature for 2 h. The sections were incubated with 1:100 rabbit polyclonal primary antibody against ferroportin-1 (catalog no. PA5-22993; Invitrogen, Thermo Scientific, MA, USA) in blocking solution at 4 °C overnight. The primary antibody was omitted for the negative control. After that, the sections were washed and incubated with 1:100 goat anti-rabbit conjugated FITC (catalog no. 111-095-003; Jackson Immuno Research, PA, USA) in blocking solution at room temperature in a dark condition for 1 h. After washing, the sections were incubated with Vector TrueVIEW reagent for 5 min, then washed out. The sections were mounted with VECTASHIELD Vibrance with DAPI (catalog no. SP-8500; Vector Laboratories, Inc., Newark, CA). Ferroportin-1 protein expression in the duodenum was visualized and captured by a confocal laser-scanning microscope (model Zeiss LSM800; Carl Zeiss AG, Germany).

Molecular docking of mouse ferroportin-1 protein and VIT-2763

The structure of mouse ferroportin-1 was obtained from the AlphaFold Protein Structure Database. The initial VIT-2763-binding pocket was predicted using AutoDock Vina, in which the ligand was docked onto the AlphaFold-derived ferroportin-1 model to identify the most favorable binding poses. Binding site comparisons for VIT-2763 and hepcidin were performed using structural information from the cryo-EM complexes 8BZY and 6WBV, respectively, retrieved from the Protein Data Bank (https://www.rcsb.org/). The structural visualization was performed

using Chimera [5] and Pymol Molecular Graphics System (Schrödinger, LLC). 2D plot of protein-ligand interaction was generated using PoseView [6].

REFERENCES

1. Charoenphandhu N, Aeimlapa R, Sooksawanwit S, Thongbunchoo J, Teerapornpuntakit J, Svasti S, et al. Responses of primary osteoblasts and osteoclasts from hemizygous β-globin knockout thalassemic mice with elevated plasma glucose to 1,25-dihydroxyvitamin D_3. Sci Rep. 2019;9:13963.
2. Stanford CM, Jacobson PA, Eanes ED, Lembke LA, Midura RJ. Rapidly forming apatitic mineral in an osteoblastic cell line (UMR 106-01 BSP). J Biol Chem. 1995;270:9420–8.
3. Alatalo SL, Halleen JM, Hentunen TA, Monkkonen J, Vaananen HK. Rapid screening method for osteoclast differentiation *in vitro* that measures tartrate-resistant acid phosphatase 5b activity secreted into the culture medium. Clin Chem. 2000;46:1751–4.
4. Araujo JC, Poblenz A, Corn P, Parikh NU, Starbuck MW, Thompson JT, et al. Dasatinib inhibits both osteoclast activation and prostate cancer PC-3-cell-induced osteoclast formation. Cancer Biol Ther. 2009;8:2153–9.
5. Pettersen EF, Goddard TD, Huang CC, Couch GS, Greenblatt DM, Meng EC, et al. UCSF Chimera--a visualization system for exploratory research and analysis. J Comput Chem. 2004;25:1605–12.
6. Stierand K RM. PoseView -- molecular interaction patterns at a glance. J Cheminform. 2010;4:P50.
