# Supplementary material for: VIT-2763, a ferroportin-1 inhibitor, modulates osteoblast and osteoclast functions in hemizygous βIVSII-654 knock-in thalassemic mice with osteopenia
Source: Cell Death Discov. 2026 Jul 31;12:429. doi: 10.1038/s41420-026-03273-x (PMC13714295; doi:10.1038/s41420-026-03273-x)
Supplement: Supplementary file 2 [file 41420_2026_3273_MOESM2_ESM.pdf]

*Supplementary Table S1–S3*

ORIGINAL ARTICLE

# VIT-2763, a ferroportin-1 inhibitor, modulates osteoblast and osteoclast functions in hemizygous $\beta^{IVSII\text{-}654}$ knock-in thalassemic mice with osteopenia

Ratchaneevan Aeimlapa[1,2], Jarinthorn Teerapornpuntakit[1,3], Jirawan Thongbunchoo[1,2], Pathnaree Wattano[1], Jiraporn Panmanee[4], Saovaros Svasti[5], Narattaphol Charoenphandhu[1,2,6,7,*]

[1] Center of Calcium and Bone Research (COCAB), Faculty of Science, Mahidol University, Bangkok, Thailand
[2] Department of Physiology, Faculty of Science, Mahidol University, Bangkok, Thailand
[3] Physiology Division, Preclinical Science, Faculty of Medicine, Thammasat University, Pathum Thani, Thailand
[4] Research Center for Neuroscience, Institute of Molecular Biosciences, Mahidol University, Nakhon Pathom, Thailand
[5] Thalassemia Research Center, Institute of Molecular Biosciences, Mahidol University, Nakhon Pathom, Thailand
[6] Institute of Molecular Biosciences, Mahidol University, Nakhon Pathom, Thailand
[7] The Academy of Science, The Royal Society of Thailand, Bangkok, Thailand

**Running title:** Anti-resorptive effects of ferroportin-1 inhibitor

*** Corresponding author**

Narattaphol Charoenphandhu, M.D., Ph.D.
Department of Physiology
Faculty of Science, Mahidol University
Rama VI Road, Bangkok 10400
Thailand
E-mail: naratt@narattsys.com

**Keywords:** β-thalassemia; vamifeport (VIT-2763); three-dimensional (3D) holotomography; osteoblast; osteoclast

**Supplementary Table S1.** *Mus musculus* primers for real-time PCR

| Gene | Accession no. | Primer (Forward/Reverse) | Product size (bp) | Annealing temperature (°C) |
|---|---|---|---|---|
| *Osteoblast differentiation markers* | | | | |
| Runx2 | NM_001146038.2 | 5′-AGGGACTATGGCGTCAAACA-3′<br>5′-GGCTCACGTCGCTCATCTT-3′ | 137 | 57 |
| ALP | NM_001287172.1 | 5′-TCTCCAGACCCTGCAACCTC-3′<br>5′-CATCCTGAGCAGACCTGGTC-3′ | 150 | 58 |
| Osteocalcin | NM_001305448.1 | 5′-ACGGTATCACTATTTAGGACCTGTG-3′<br>5′-ACTTTATTTTGGAGCTGCTGTGAC-3′ | 141 | 57 |
| Osteopontin | NM_001204203.1 | 5′-TGTGGACTCGGATGAATCTGAC-3′<br>5′-CGACTGTAGGGACGATTGGAG-3′ | 107 | 59 |
| *Osteoblast-derived osteoclastogenic factors* | | | | |
| RANKL | NM_011613.3 | 5′-CGCTCTGTTCCTGTACTTTCG-3′<br>5′-CTCTCCAGAGTCGAGTCCTGC-3′ | 126 | 57 |
| M-CSF | NM_007778.4 | 5′-GACCCTCGAGTCAACAGAGC-3′<br>5′-TGTCAGTCTCTGCCTGGATG-3′ | 236 | 58 |
| *Osteoclast differentiation markers* | | | | |
| RANK | NM_009399.3 | 5′-CATCTTCGGCGTTTACTACAGG-3′<br>5′-TCCACTTAGACTACTGCAAGCA-3′ | 91 | 57 |
| Calcitonin receptor (Calr) | NM_001355192.1 | 5′-CGGACTTTGACACAGCAGAA-3′<br>5′-GTCACCCTCTGGCAGCTAAG-3′ | 247 | 55 |
| Cathepsin K (CTSK) | NM_007802.4 | 5′-GAACGAGAAAGCCCTGAAGAGA-3′<br>5′-TATCGAGTGCTTGCTTCCCTTC-3′ | 190 | 57 |
| Atp6v0d2 | NM_175406.3 | 5′-GACCCTGTGGCACTTTTTGT-3′<br>5′-GTGTTTGAGCTTGGGGAGAA-3′ | 102 | 57 |
| NFATc1 | NM_001164112.1 | 5′-CCGTTGCTTCCAGAAAATAACA-3′<br>5′-TGTGGGATGTGAACTCGGAA-3′ | 152 | 54 |
| *Iron transport and regulatory factors* | | | | |
| Transferrin receptor 1 (TfR1) | NM_011638.4 | 5′-TCGCTTATATTGGGCAGACC-3′<br>5′-TGACCAATGCTGCTTTTCAC-3′ | 234 | 57 |
| Dcytb | NM_028593.2 | 5′-ATGTACAGCCTGCACAGC-3′<br>5′-TGTCACTCCCATGAGAACC-3′ | 192 | 56 |
| DMT1 | NM_001146161.1 | 5′-TCAGAGCTCCACCATGACTG-3′<br>5′-TGTGAACGTGAGGATGGGTA-3′ | 235 | 57 |
| Ferritin | NM_010239.2 | 5′-AAGTGCGCCAGAACTACCAC-3′<br>5′-TGGTTCTGCAGCTTCATCAG-3′ | 205 | 57 |
| Ferropontin-1 (FPN1) | NM_016917.2 | 5′-CTACCATTAGAAGGATTGACCAGCTA-3′<br>5′-ACTGGAGAACCAAATGTCATAATCTG-3′ | 82 | 56 |
| Nrf2 | NM_010902.5 | 5′-AGTCTTCACTGCCCCTCATC-3′<br>5′-AACAGCGGTAGTATCAGCCA-3′ | 175 | 58 |
| *Cell cycle- and apoptosis-related genes* | | | | |
| Cyclin D1 | NM_001379248.1 | 5′-CAGAAGTGCGAAGAGGAGGTC-3′<br>5′-TCATCTTAGAGGCCACGAACAT-3′ | 130 | 56 |
| Cdkn2a | NM_009877.2 | 5′-ACGTTCACGTAGCAGCTCTT-3′<br>5′-TCTTGATGTCCCCGCTCTTG-3′ | 208 | 58 |
| Cdkn1a | NM_001111099.2 | 5′-GGTGGGCTTATCTGGGATGG-3′<br>5′-ATGTTTGGGGCTGGAGTCAG-3′ | 207 | 58 |
| Bcl-2 | NM_009741.5 | 5′-ATGCCTTTGTGGAACTATATGGC-3′<br>5′-GGTATGCACCCAGAGTGATGC-3′ | 120 | 56 |
| Caspase-3 | NM_009810.3 | 5′-CCTCAGAGAGACATTCATGG-3′<br>5′-GCAGTAGTCGCCTCTGAAGA-3′ | 226 | 55 |

| Gene | Accession no. | Primer (Forward/Reverse) | Product size (bp) | Annealing temperature (°C) |
|---|---|---|---|---|
| Caspase-9 | NM_001429869.1 | 5′-CAGGACCTTGGACAGTGACT-3′<br>5′-AATGCCATCCAAGGTCTCGA-3′ | 196 | 58 |
| Cytochrome c | NM_007808.5 | 5′-GAGGCAAGCATAAGACTGGA-3′<br>5′-TACTCCATCAGGGTATCCTC-3′ | 133 | 55 |
| *Mitophagy-related genes* | | | | |
| Beclin 1 | NM_019584.4 | 5′-ACCGGGAAGTAGCTGAAGAC-3′<br>5′-AGCTCGTGTCCAGTTTCAGA-3′ | 210 | 58 |
| Pink1 | NM_026880.2 | 5′-TATAGGAAAGGGCCCGGATG-3′<br>5′-GCTCCATACTCTCCAGCCAA-3′ | 192 | 59 |
| *Housekeeping genes* | | | | |
| GAPDH | NM_008084.2 | 5′-TTCCAGTATGACTCCACTCACG-3′<br>5′-AAGACACCAGTAGACTCCACGA-3′ | 167 | 55 |
| β-actin | NM_007393.3 | 5′-CCAGGTCATCACTATTGGCA-3′<br>5′-ACCACCAGACAGCACTGTGTT-3′ | 172 | 54 |
| 18S rRNA | NR_003278.3 | 5′-GTAACCCGTTGAACCCCATT-3′<br>5′-CCATCCAATCGGTAGTAGCG-3′ | 151 | 57 |

Runx2, *runt*-related transcription factor-2; ALP, alkaline phosphatase; RANKL, receptor activator of nuclear factor-κB ligand; M-CSF, macrophage colony-stimulating factor; RANK, receptor for receptor activator of nuclear factor-κB ligand; Calr, Calcitonin receptor; CTSK, cathepsin K; Atp6V0d2, ATPase $H^+$ Transporting V0 Subunit D2; NFATc1, nuclear factor of activated T-cells c1; Dcytb, duodenal cytochrome b; DMT1, divalent metal transporter 1; FPN1, ferroportin-1; Nrf2, nuclear factor erythroid 2-related factor 2; Cdkn2a, cyclin dependent kinase inhibitor 2A; Cdkn1a, cyclin dependent kinase inhibitor 1A; Bcl-2, B-cell lymphoma-2; Pink1, PTEN-induced kinase 1

**Supplementary Table S2.** Complete blood count (CBC)

| Parameter | WT | B654 |
|---|---|---|
| RBC ($10^6$/μL) | 11.14 ± 0.317 | 9.945 ± 0.277 $^{*}$ |
| HGB (g/dL) | 13.93 ± 0.501 | 9.675 ± 0.236 $^{***}$ |
| HCT (%) | 47.60 ± 1.196 | 32.13 ± 0.455 $^{***}$ |
| MCV (fL) | 42.50 ± 0.289 | 32.25 ± 0.629 $^{***}$ |
| MCH (pg) | 12.53 ± 0.180 | 9.750 ± 0.323 $^{***}$ |
| MCHC (g/dL) | 29.25 ± 0.427 | 30.10 ± 0.480 |
| RDW-SD (fL) | 31.80 ± 0.200 | 46.28 ± 1.028 $^{***}$ |

Data expressed as mean ± SE ($n$ = 4). RBC, red blood cell; HGB, hemoglobin; HCT, hematocrit; MCV, mean corpuscular volume; MCH, mean corpuscular hemoglobin; MCHC, mean corpuscular hemoglobin concentration; RDW-SD, red cell distribution width-standard deviation; WT, wild-type mice; B654, hemizygous $\beta^{IVSII-654}$ knock-in thalassemic (B654) mice. *$P < 0.05$, ***$P < 0.001$ vs. WT by unpaired one-tailed Student's $t$ test.

**Supplementary Table S3.** Cellular morphology and properties

| **Cellular property** | **20 µM FAC + Vehicle** | **20 µM FAC + 2 µM VIT-2763** | **20 µM FAC + 20 µM VIT-2763** |
|---|---|---|---|
| Volume (µm$^3$) | 12476 ± 2252 | 17005 ± 5103 | 12693 ± 3736 |
| Surface area (µm$^2$) | 8129 ± 992.9 | 10285 ± 2800 | 7674 ± 1683 |
| Projected area (µm$^2$) | 1782 ± 234.9 | 2142 ± 571.7 | 1648 ± 357.9 |
| Sphericity | 0.3171 ± 0.019 | 0.3248 ± 0.046 | 0.3458 ± 0.026 |
| Dry mass (pg) | 569.3 ± 120.2 | 1133 ± 298.0 | 919.0 ± 241.7 |

Data expressed as mean ± SE ($n$ = 4–9). FAC, ferric ammonium citrate.
