# Supplementary material for: VIT-2763, a ferroportin-1 inhibitor, modulates osteoblast and osteoclast functions in hemizygous βIVSII-654 knock-in thalassemic mice with osteopenia
Source: Cell Death Discov. 2026 Jul 31;12:429. doi: 10.1038/s41420-026-03273-x (PMC13714295; doi:10.1038/s41420-026-03273-x)
Supplement: Supplementary file 3 [file 41420_2026_3273_MOESM3_ESM.pdf]

Supplementary Materials

Supplementary Figures S1–S5

ORIGINAL ARTICLE

VIT-2763, a ferroportin-1 inhibitor, modulates osteoblast and osteoclast functions in hemizygous $\beta^{IVSII-654}$ knock-in thalassemic mice with osteopenia

Ratchaneevan Aeimlapa[1,2], Jarinthorn Teerapornpuntakit[1,3], Jirawan Thongbunchoo[1,2], Pathnaree Wattano[1], Jiraporn Panmanee[4], Saovaros Svasti[5], Narattaphol Charoenphandhu[1,2,6,7,*]

[1] Center of Calcium and Bone Research (COCAB), Faculty of Science, Mahidol University, Bangkok, Thailand
[2] Department of Physiology, Faculty of Science, Mahidol University, Bangkok, Thailand
[3] Physiology Division, Preclinical Science, Faculty of Medicine, Thammasat University, Pathum Thani, Thailand
[4] Research Center for Neuroscience, Institute of Molecular Biosciences, Mahidol University, Nakhon Pathom, Thailand
[5] Thalassemia Research Center, Institute of Molecular Biosciences, Mahidol University, Nakhon Pathom, Thailand
[6] Institute of Molecular Biosciences, Mahidol University, Nakhon Pathom, Thailand
[7] The Academy of Science, The Royal Society of Thailand, Bangkok, Thailand

Running title: Anti-resorptive effects of ferroportin-1 inhibitor

*** Corresponding author**

Narattaphol Charoenphandhu, M.D., Ph.D.
Department of Physiology
Faculty of Science, Mahidol University
Rama VI Road, Bangkok 10400
Thailand
E-mail: naratt@narattsys.com

Keywords: β-thalassemia; vamifeport (VIT-2763); three-dimensional (3D) holotomography; osteoblast; osteoclast

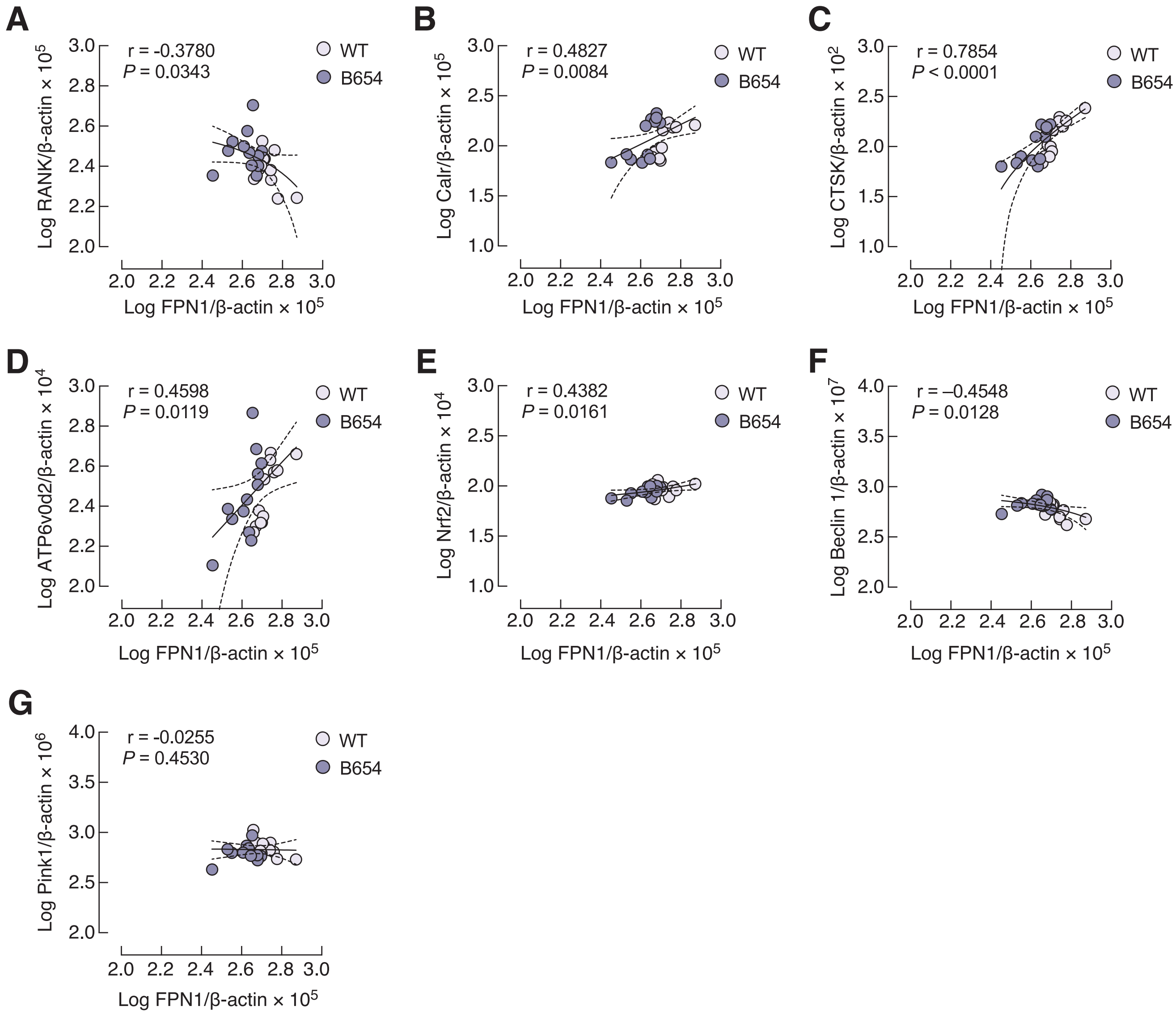

Supplementary Figure S1: Correlation plot between mRNA expression of ferroportin-1 and osteoclast-, Nrf2-, and mitophagy-related genes. Correlation between mRNA expression of ferroportin-1 and **A** receptor for receptor activator of nuclear factor-κB ligand (RANK), **B** calcitonin receptor (Calr), **C** cathepsin K (CTSK), **D** vacuolar ATPase H^+ transporting V0 subunit D2 (ATP6v0d2 or V-ATPase), transcription factor, i.e., **E** nuclear factor erythroid 2-related factor 2 (Nrf2), and mitophagy-related genes, i.e., **F** Beclin 1, **G** PTEN-induced kinase 1 (Pink1) in primary osteoclasts derived from wild-type (WT) and hemizygous $\beta^{IVSII-654}$ knock-in thalassemic (B654) mice (*n* = 12). Dashed line represents the 95% confident bands of the best-fit line. r Pearson correlation coefficient. *P* p-value of the correlation.

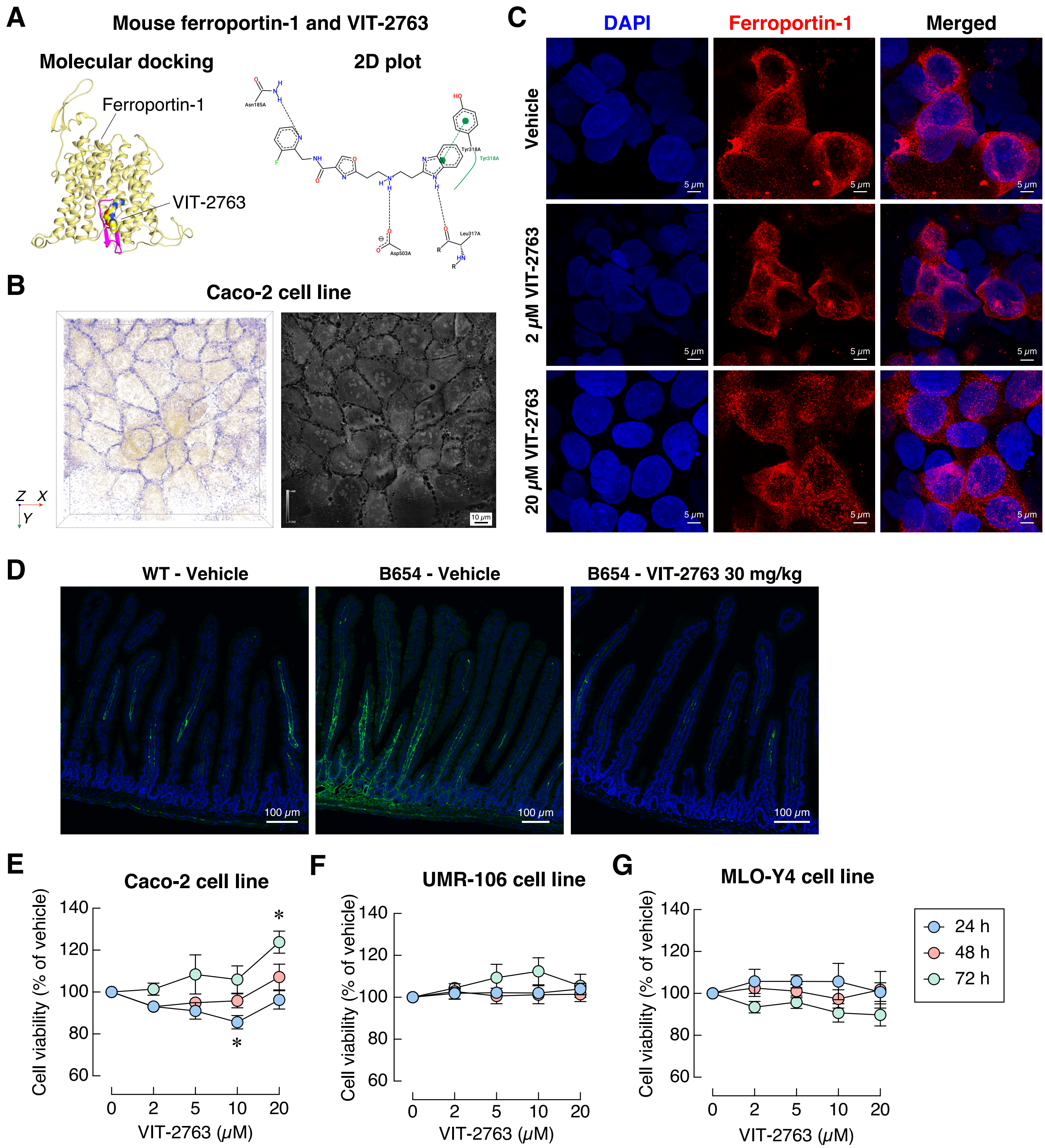

Supplementary Figure S2: Effective reduction of ferroportin-1 protein expression in intestinal cells by VIT-2763. A Molecular docking and 2D plot of mouse ferroportin-1 protein and VIT-2763 interaction. **B** Representative three-dimentional (3D) hologram images of Caco-2 monolayer. Scale bars, 10 µm (×40 magnification). Immunofluorescence staining of ferroportin-1 protein in **C** Caco-2 monolayer after exposure to 2, 20 µM VIT-2763 for 18 h and **D** duodenums isolated from wild-type (WT) and hemizygous $\beta^{IVSII-654}$ knock-in thalassemic (B654) mice after oral administration of 30 mg/kg VIT-2763 daily for 3 days. The images were captured by confocal microscope. Scale bars, 5 µm (×100 magnification) for Caco-2 cells, and 100 µm (×10 magnification) for duodenums. Cell viability of **E** Caco-2, **F** UMR-106, **G** MLO-Y4 cell line after exposure to 2, 5, 10, 20 µM VIT-2763 for 24, 48, and 72 h (n = 4–6 independent experiments, triplicate/concentration). Data expressed as mean ± SE. *P < 0.05 vs. vehicle-treated group (0 µM) at the same timepoint by one-way analysis of variance (ANOVA) with Dunnett's multiple comparisons test.

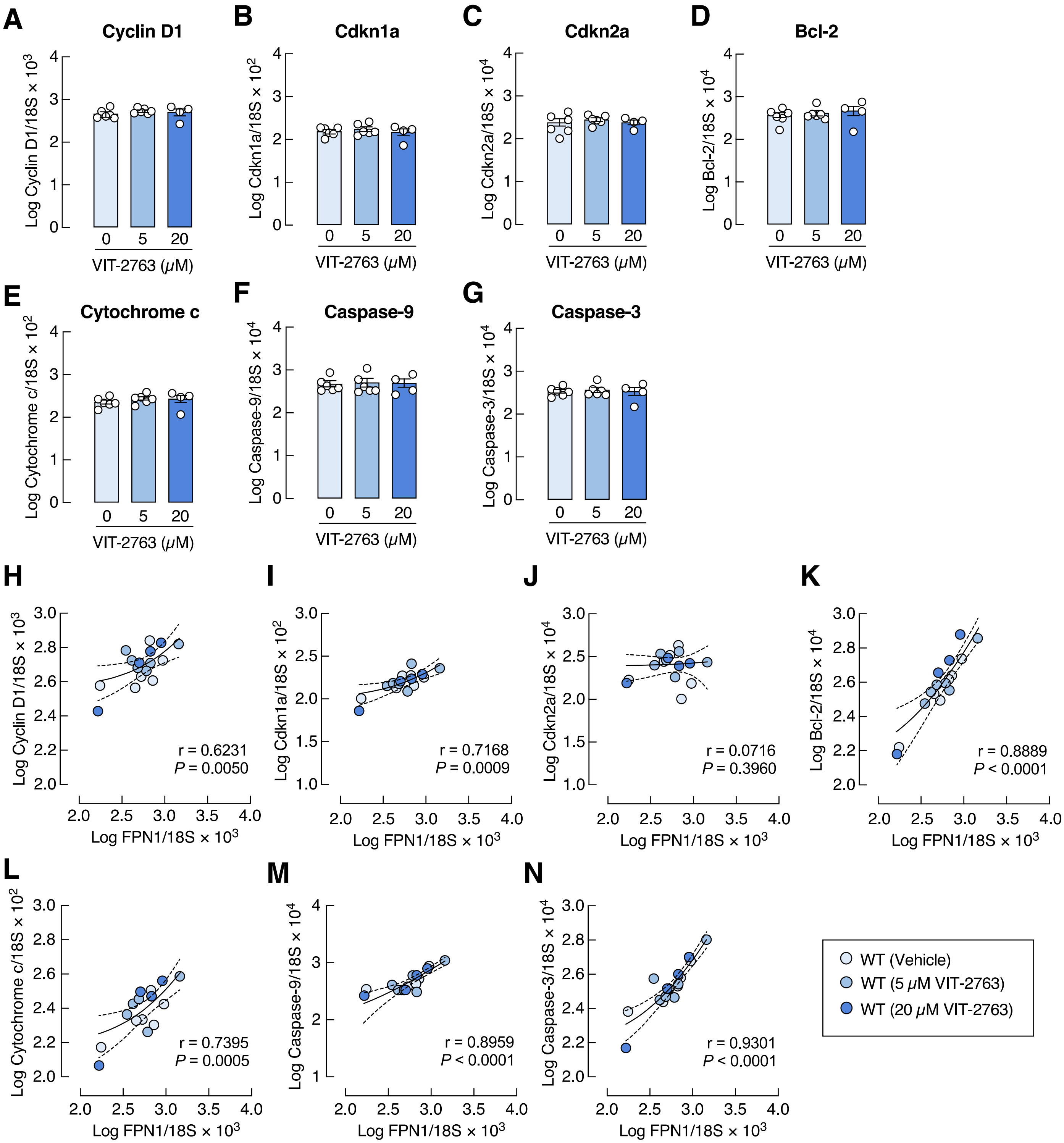

Supplementary Figure S3: Unaltered mRNA expression of cell cycle- and cell apoptosis-related genes following VIT-2763 treatment. mRNA expression of cell cycle-related genes, i.e., **A** cyclin D1, **B** cyclin-dependent kinase inhibitor 1A (Cdkn1a; p21), **C** cyclin-dependent kinase inhibitor 2A (Cdkn2a), **D** a key regulator of apoptosis Bcl-2, cell apoptossis-related genes, i.e., **E** cytochrome c, **F** caspase-9, **G** caspase-3 in wild-type (WT)-derived primary osteoblasts after 5, 20 µM VIT-2763 treatment. Data expressed as mean ± SE (*n* = 4–6). Correlation plot between mRNA expression of ferroportin-1 and **H** cyclin D1, **I** Cdkn1a, **J** Cdkn2a, **K** Bcl-2, **L** cytochrome c, **M** caspase-9, **N** caspase-3 in WT-derived primary osteoblasts. Dashed line represents the 95% confident bands of the best-fit line. r Pearson correlation coefficient. *P* p-value of the correlation.

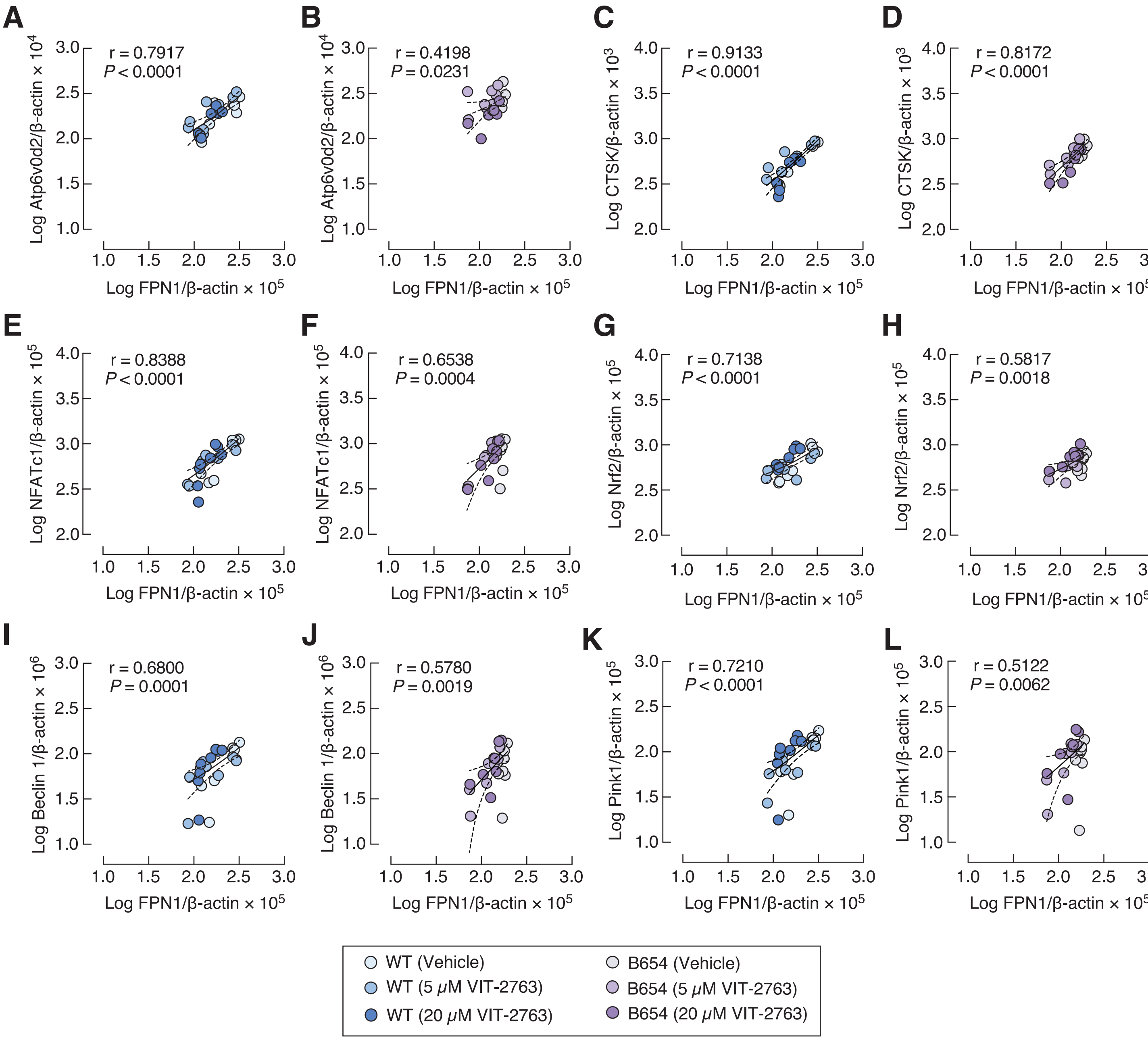

Supplementary Figure S4: Positive correlation between ferroportin-1 mRNA expression and osteoclast-, Nrf2-, and mitophagy-related genes. Correlation plot between mRNA expression of ferroportin-1 and osteoclast-specific genes, i.e., **A–B** vacuolar ATPase H^+ transporting V0 subunit D2 (ATP6v0d2 or V-ATPase), **C–D** cathepsin K (CTSK), signaling factor, i.e., **E–F** nuclear factor of activated T-cells c1 (NFATc1), transcription factor, i.e., **G–H** nuclear factor erythroid 2-related factor 2 (Nrf2), and mitophagy-related genes, i.e., **I–J** Beclin 1, **K–L** PTEN-induced kinase 1 (Pink1) in primary osteoclasts derived from bone marrow cells of **A, C, E, G, I, K** wild-type (WT) and **B, D, F, H, J, L** hemizygous $\beta^{IVSII-654}$ knock-in thalassemic (B654) mice after treated with 5, 20 μM VIT-2763 for 3 days (*n* = 7–8). Dashed line represents the 95% confident bands of the best-fit line. r Pearson correlation coefficient. *P* p-value of the correlation.

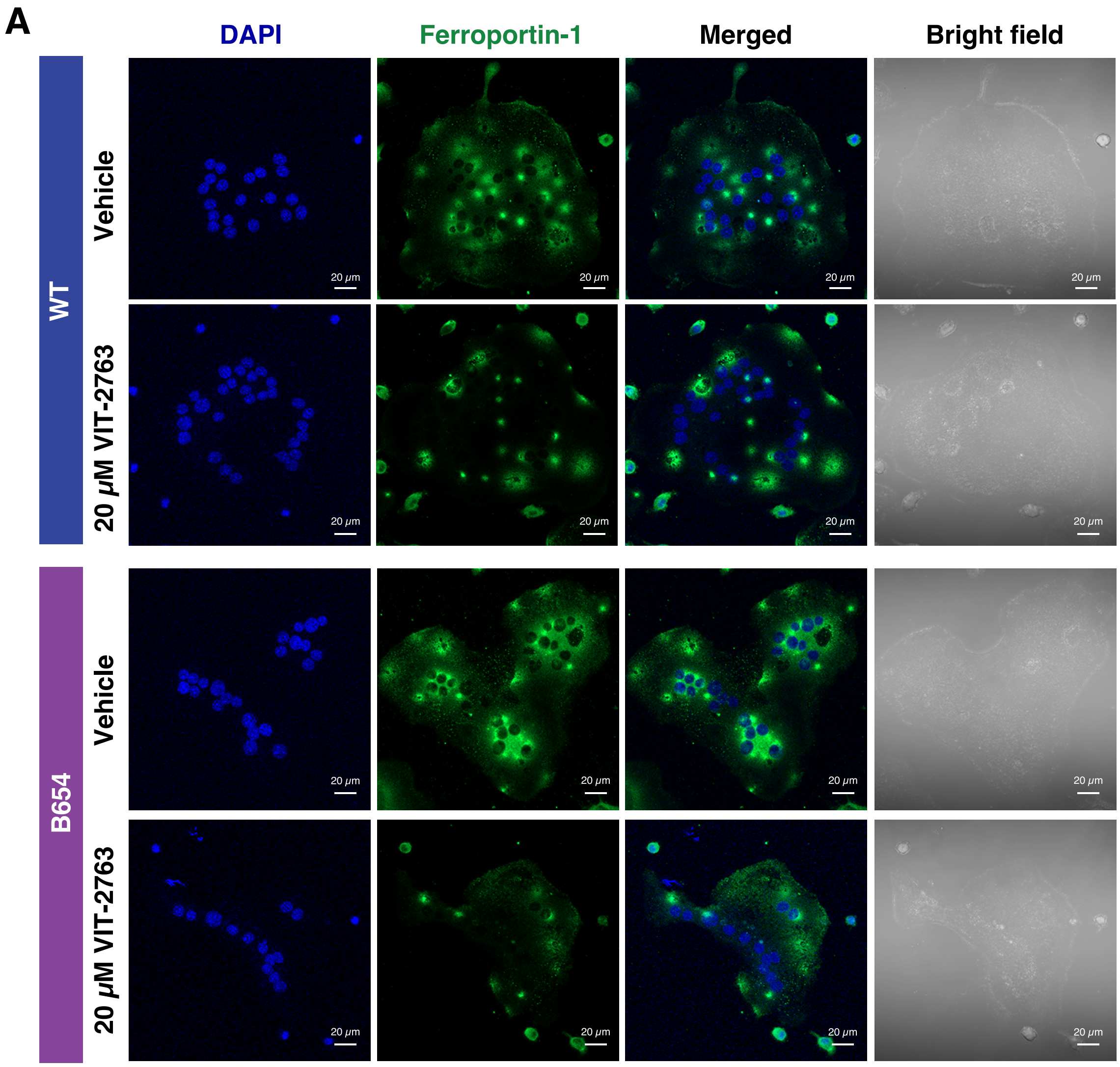

Supplementary Figure S5: Attenuation of ferroportin-1 and V-ATPase protein expression in primary osteoclasts by VIT-2763. Immunofluorescence staining of **A** ferroportin-1 protein in primary osteoclasts derived from bone marrow cells of wild-type (WT) and hemizygous $\beta^{IVSII\text{-}654}$ knock-in thalassemic (B654) mice after treated with 20 μM VIT-2763 for 3 days. The images were captured by confocal microscope. Scale bars, 20 μm (×40 magnification).

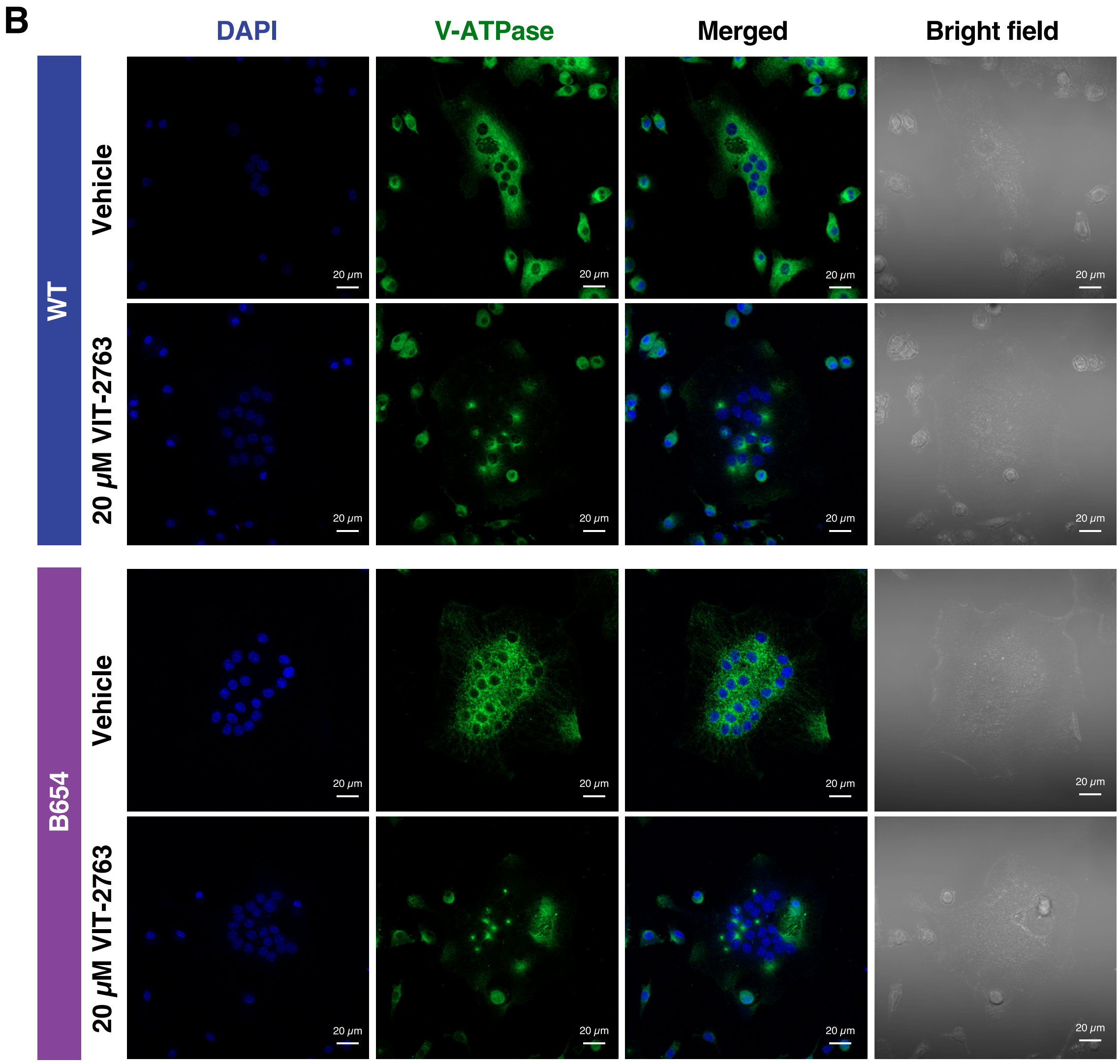

Supplementary Figure S5 (cont.): Attenuation of ferroportin-1 and V-ATPase protein expression in primary osteoclasts by VIT-2763. Immunofluorescence staining of **B** vacuolar ATPase H^+ transporting V0 subunit D2 (ATP6v0d2 or V-ATPase) protein in primary osteoclasts derived from bone marrow cells of wild-type (WT) and hemizygous $\beta^{IVSII\text{-}654}$ knock-in thalassemic (B654) mice after treated with 20 µM VIT-2763 for 3 days. The images were captured by confocal microscope. Scale bars, 20 µm (×40 magnification).
