# Supplementary material for: VIT-2763, a ferroportin-1 inhibitor, modulates osteoblast and osteoclast functions in hemizygous βIVSII-654 knock-in thalassemic mice with osteopenia
Source: Cell Death Discov. 2026 Jul 31;12:429. doi: 10.1038/s41420-026-03273-x (PMC13714295; doi:10.1038/s41420-026-03273-x)
Supplement: Supplementary file 4 [file 41420_2026_3273_MOESM5_ESM.pdf]

Supplementary Materials

Supplementary Video Legend

ORIGINAL ARTICLE

VIT-2763, a ferroportin-1 inhibitor, modulates osteoblast and osteoclast functions in hemizygous $\beta^{IVSII-654}$ knock-in thalassemic mice with osteopenia

Ratchaneevan Aeimlapa[1,2], Jarinthorn Teerapornpuntakit[1,3], Jirawan Thongbunchoo[1,2], Pathnaree Wattano[1], Jiraporn Panmanee[4], Saovaros Svasti[5], Narattaphol Charoenphandhu[1,2,6,7,*]

[1] Center of Calcium and Bone Research (COCAB), Faculty of Science, Mahidol University, Bangkok, Thailand
[2] Department of Physiology, Faculty of Science, Mahidol University, Bangkok, Thailand
[3] Physiology Division, Preclinical Science, Faculty of Medicine, Thammasat University, Pathum Thani, Thailand
[4] Research Center for Neuroscience, Institute of Molecular Biosciences, Mahidol University, Nakhon Pathom, Thailand
[5] Thalassemia Research Center, Institute of Molecular Biosciences, Mahidol University, Nakhon Pathom, Thailand
[6] Institute of Molecular Biosciences, Mahidol University, Nakhon Pathom, Thailand
[7] The Academy of Science, The Royal Society of Thailand, Bangkok, Thailand

Running title: Anti-resorptive effects of ferroportin-1 inhibitor

*** Corresponding author**

Narattaphol Charoenphandhu, M.D., Ph.D.
Department of Physiology
Faculty of Science, Mahidol University
Rama VI Road, Bangkok 10400
Thailand
E-mail: naratt@narattsys.com

Keywords: β-thalassemia; vamifeport (VIT-2763); three-dimensional (3D) holotomography; osteoblast; osteoclast

Video Legend

Video S1: Apoptosis during full osteoclast differentiation. Representative time-lapse video of label-free live cell imaging of primary osteoclasts. Time-lapse holotomograms were continuously captured every 20 min for 8 h during day 6 to 7 of culture. The images were captured by a holotomographic microscope.
